# Recommended approaches for integration of population pharmacokinetic modelling with precision dosing in clinical practice

**DOI:** 10.1111/bcp.16335

**Published:** 2024-11-21

**Authors:** Monika Berezowska, Isaac S. Hayden, Andrew M. Brandon, Arsenii Zats, Mehzabin Patel, Shelby Barnett, Kayode Ogungbenro, Gareth J. Veal, Alaric Taylor, Jugal Suthar

**Affiliations:** ^1^ Vesynta Ltd, Innovation Gateway, The London Cancer Hub, Cotswold Road, Sutton London UK; ^2^ Translational and Clinical Research Institute Newcastle University Centre for Cancer Newcastle upon Tyne UK; ^3^ Division of Pharmacy & Optometry, School of Health Sciences University of Manchester Manchester UK

**Keywords:** Modelling, pharmacokinetics, pharmacometrics, simulation prescribing, therapeutic drug monitoring

## Abstract

Current methods of dose determination have contributed to suboptimal and inequitable health outcomes in underrepresented patient populations. The persistent demand to individualise patient treatment, alongside increasing technological feasibility, is leading to a growing adoption of model‐informed precision dosing (MIPD) at point of care. Population pharmacokinetic (popPK) modelling is a technique that supports treatment personalisation by characterising drug exposure in diverse patient groups. This publication addresses this important shift in clinical approach, by collating and summarising recommendations from literature. It seeks to provide standardised guidelines on best practices for the development of popPK models and their use in MIPD software tools, ensuring the safeguarding and optimisation of patient outcomes. Moreover, it consolidates guidance from key regulatory and advisory bodies on MIPD software deployment, as well as technical requirements for electronic health record integration. It also considers the future application and clinical impact of machine learning algorithms in popPK and MIPD. Ultimately, this publication aims to facilitate the incorporation of high‐quality precision‐dosing solutions into standard clinical workflows, thereby enhancing the effectiveness of individualised dose selection at point of care.

**What is already known about this subject**

- In some drug–disease combinations, current methods of dose determination can lead to suboptimal health outcomes in underrepresented patient populations.
- There is an increasing proliferation of model‐informed precision dosing (MIPD) software platforms using population pharmacokinetic models to individualise patient treatments to address *1‐size‐fits‐all* dosing approaches.

**What this review adds**

- Best practice‐led recommendations on the development and validation of population pharmacokinetic models, and their effective deployment in MIPD software platforms to optimise patient treatment regimens as part of standard clinical care.
- Guidance from key regulatory and advisory bodies on MIPD software implementation, integration with electronic health records and current status of machine learning applications.

## 1 INTRODUCTION

In clinical practice, 1‐size‐fits‐all approaches to drug treatment can lead to inconsistent and adverse health outcomes due to inherent variability in individual patient characteristics.^1^ Consequently, there is a growing emphasis on treatment personalisation, such as precision dosing, to replace traditional methods of dose selection.^2^

For many drugs, a fixed adult dose is adequate,^1^ where the drug has a wide therapeutic index and minimal toxicity profile. Otherwise, dose adjustments are often made based on a single factor such as body weight,^3^ body surface area^4^ or renal function.^5^ However, this does not sufficiently account for variabilities, such as between‐subject variability (BSV), and results in challenging patient subpopulations (such as paediatrics, pregnant women and the obese) being underrepresented and potentially placed at clinical risk.^1^, ^3^

Individualised dose optimisation can provide a solution where a drug has a known exposure–response/exposure–safety relationship, a narrow therapeutic index, is dosed near the maximum tolerated dose, exhibits wide BSV, or is licensed for high‐risk patient populations.^6^ Individualising the dose is particularly important where the observed variability between patients, which is not explained by their covariate characteristics, is larger than the assumed safe and effective variability threshold, but can be determined using a drug's therapeutic margin.^7^ Optimisation may be achieved through a therapeutic drug monitoring (TDM)^2^, ^8^ approach to treatment adjustment, either in its traditional form or by target concentration intervention (TCI),^9^ often considering drug pharmacokinetics (PK) and/or pharmacodynamics (PD).

Traditional TDM compares patient plasma or blood drug concentrations against defined therapeutic windows, informing a dose adjustment at the clinician's discretion.^9^ It requires a quantifiable relationship between administered drug dose and circulating concentrations.^8^ TDM has been used to reduce dose‐related toxicity for numerous drugs, historically including theophylline and phenytoin, and more recently busulfan, vancomycin, tacrolimus and methotrexate,^10^, ^11^, ^12^, ^13^, ^14^ but exhibits several limitations.^9^, ^15^, ^16^

Sampling must be performed within specific time windows which can delay dose adjustments, particularly for drugs where this time window is at steady state.^15^ Additionally, when only a single sample is taken, this may not adequately describe BSV in overall drug exposure (i.e. area under the plasma drug concentration *vs*. time curve [AUC]).^15^, ^17^ Traditional TDM assumes that a measured drug concentration is *therapeutic* whenever it falls within the defined therapeutic window, but in reality concentrations within the range may still lead to suboptimal outcomes for some individuals.^15^, ^16^, ^18^ Some of these limitations have prompted an increased uptake of TCI.^9^

TCI uses specific drug concentration or biomarker targets, rather than a broad target therapeutic window.^9^ Using pharmacological principles, the dose required to achieve the target is predicted based on previous patient measurements of circulating analyte levels.^9^, ^16^ TCI has been shown to improve patient outcomes compared to traditional TDM by providing dose predictions that better account for BSV and lead to more accurate attainment of target drug exposure.^9^, ^16^

Modern approaches to dose individualisation—whether labelled as TDM or TCI—regularly involve the use of mathematical models. This process is known as model‐informed precision dosing (MIPD),^2^, ^15^ previously defined as “an advanced quantitative approach focusing on individualised dosage optimisation, integrating complex mathematical and statistical models of drugs and disease combined with individual demographic and clinical patient characteristics”.^19^ Patient characteristics (covariates) are combined with a drug‐specific population PK (popPK) or PK‐PD model to provide informed dosing recommendations for the individual.^2^ This approach maximises the chance of a given dose achieving PK and/or PD targets, resulting in an improved benefit‐to‐harm ratio.^2^, ^15^, ^20^ MIPD can simultaneously consider multiple covariates and other sources of BSV and error, thus providing a more informed dosing decision.^2^, ^15^, ^16^ PopPK can be used where there is a known relationship between drug exposure and response, and where the model has been trained on a population that represents the target patients.^21^, ^22^ PopPK employs nonlinear mixed‐effects (NLME) models due to their efficiency at handling sparse data^23^ and ability to account for both explainable and random sources of variability,^23^, ^24^ which makes them suitable for MIPD in clinical settings.

The adoption of such techniques to maximise MIPD impact is driven by industry initiatives including Project Optimus and the Model‐Informed Drug Development Pilot Programme, and clinical trials in oncology such as DETERMINE and SMPaeds.^25^, ^26^ This builds on existing practice of MIPD for drug classes such as antibiotics, anti‐infectives, antiepileptics and immunosuppressants.^15^, ^26^, ^27^ The growing acceptance of MIPD as optimal clinical practice, coupled with the proliferation of decision support software that host MIPD tools, underscores the need for comprehensive development and implementation guidelines.^2^

Currently, a variety of MIPD software offerings exist, with differing strengths and functionalities.^27^, ^28^ However, many models remain underused for MIPD because they are not designed with specific clinical applications in mind. Integrating these models into user‐friendly software can greatly enhance their impact but requires higher standards in model development, validation and usability. Expansion of these tools beyond academic and research settings has been limited despite the increasing interest in MIPD and its application to growing numbers of drug–disease combinations. This is partly due to barriers to implementation, commercialisation and regulatory compliance.^2^, ^27^, ^28^ Maximising clinical impact from MIPD software requires consideration of multiple factors, including electronic health record integration, clinical workflows, cost, product useability, patient experience, safety and efficacy.^2^, ^27^

This publication seeks to consolidate a comprehensive set of recommended practices for popPK model integration into precision dosing software for clinical applications, by considering all relevant stakeholder requirements. This will provide an end‐to‐end overview of the precision dosing workflow, including: data handling; model building, validation and reporting; selection and adaptation of pre‐existing models for MIPD software; and its application at point of care.

## 2 DATA CONSIDERATIONS

PK data used for model building may originate from multiple sources, including clinical trials with differing protocols. Crucially, model development may not be a core objective behind the PK data generation.^29^ Thus, the data may be limited or inconsistent in terms of formatting, available patient information, accuracy of sampling times, or reported measurement units.^30^, ^31^, ^32^ Formatting of data is usually required, and strategies exist for handling missing or erroneous data and potential sources of error.^32^ These considerations are summarised below, with further information on each point provided in the Supplementary Information Section 1.

### 2.1 Data collection

Establishing a data pipeline with standardisation of collection and processing ensures regulatory compliance,^33^, ^34^ reproducibility, traceability and accuracy—all critical factors for effective application in MIPD.

When prospectively planning studies, PK‐informed trial design can support the suitability of data generated for model building.^35^, ^36^, ^37^ In practice, however, optimal sample timepoints may be unfeasible due to logistical constraints. Opportunistic or scavenged sampling regimens may need to be adopted instead to reduce the burden on patients, especially in, e.g. neonatal and paediatric populations.^35^ Collection of data from specific and diverse patient subpopulations is important as it supports the building of robust models that accurately represent these patient groups and, by extension, ensures equitable access to healthcare.^38^

### 2.2 Formatting data

In 2020, the International Society of Pharmacometrics set out a recommended data structure for use in popPK modelling and noncompartmental analysis.^39^ The formatting of data for use in PK modelling largely depends on the software that will be used for model development. Previous publications have described software‐specific data formatting requirements.^24^, ^40^, ^41^

### 2.3 Data exploration and cleaning

Early visual inspection and statistical analysis can help identify patterns and potential data outliers, as well as informing the structural and statistical models.^31^, ^42^ Diagnostic tools, including statistical summaries and correlation matrices, can give an overview of covariate distributions and relationships.^31^, ^42^ More fundamentally, this process may identify unexpected, erroneous or missing data.^32^ The approach chosen for handling such data^31^, ^39^ can have significant impact on the accuracy and precision of final model estimates.^31^, ^32^ Regardless of decisions made during the data cleaning process, all processing actions should be recorded with justification, for inclusion in the model report to support validity assessment (see Section 3.4 and Supplementary Information Section 3).

## 3 MODEL BUILDING

**FIGURE 1:**
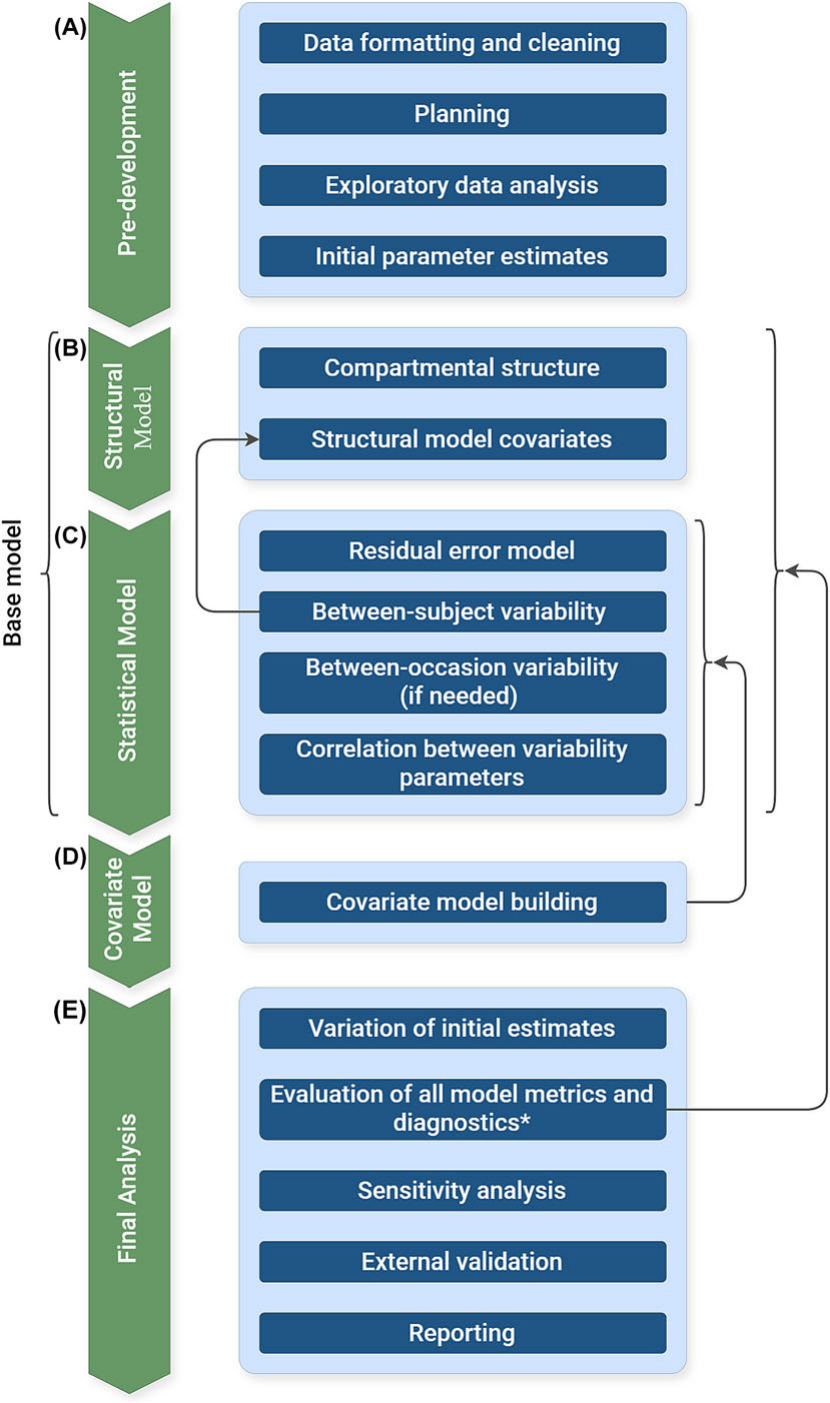
A workflow diagram of the recommended steps in the pharmacokinetic model development process: (A) Predevelopment, (B) structural model, (C) statistical model, (D) covariate model, (E) final analysis. * Appropriate metrics and diagnostics should be considered throughout the building process, but all should be considered collectively at this step. Adapted from Byon *et al.* ^31^

In 2022, the US Food and Drug Administration published updated guidance on popPK analysis for new drug applications,^43^ with recommendations ranging from data collection and analysis to model building and reporting. Other prominent publications have described this process from a more technical perspective.^24^, ^31^, ^44^, ^45^ Additionally, the pharmacometrics community share useful tools and knowledge about model development.^46^, ^47^, ^48^, ^49^, ^50^, ^51^ The recommended process of building a popPK model described in these resources is summarised in Figure 1 and briefly discussed below. Supplementary Information Section 2 contains an expanded version of Section 3, providing greater detail on the popPK model building process.

### 3.1 Planning model development

Prior to popPK model development, it is best practice to prepare a population modelling analysis plan (Figure 1A).^52^, ^53^ The population modelling analysis plan should clearly state the purpose and objectives of analysis and briefly describe the data‐originating study (if applicable), specify the modelling methodology, data handling procedures, information available prior to the study and any assumptions made by the modeller.^31^, ^52^

### 3.2 Software

The earliest software for popPK model building was NONMEM^54^; hence many available guidelines and publications are oriented around NONMEM nomenclature. Since then, numerous licensed software tools, such as Monolix, Pumas, Phoenix NLME and PoPy^55^, ^56^, ^57^, ^58^ have been developed and adopted. Moreover, open‐source R packages such as nlmixr2, posologyr, mapbayr, mrgsolve and rxode2^59^, ^60^, ^61^, ^62^, ^63^, ^64^, ^65^ have gained popularity, which either replicate, complement or augment the functionality of the privately licensed software.

**FIGURE 2:**
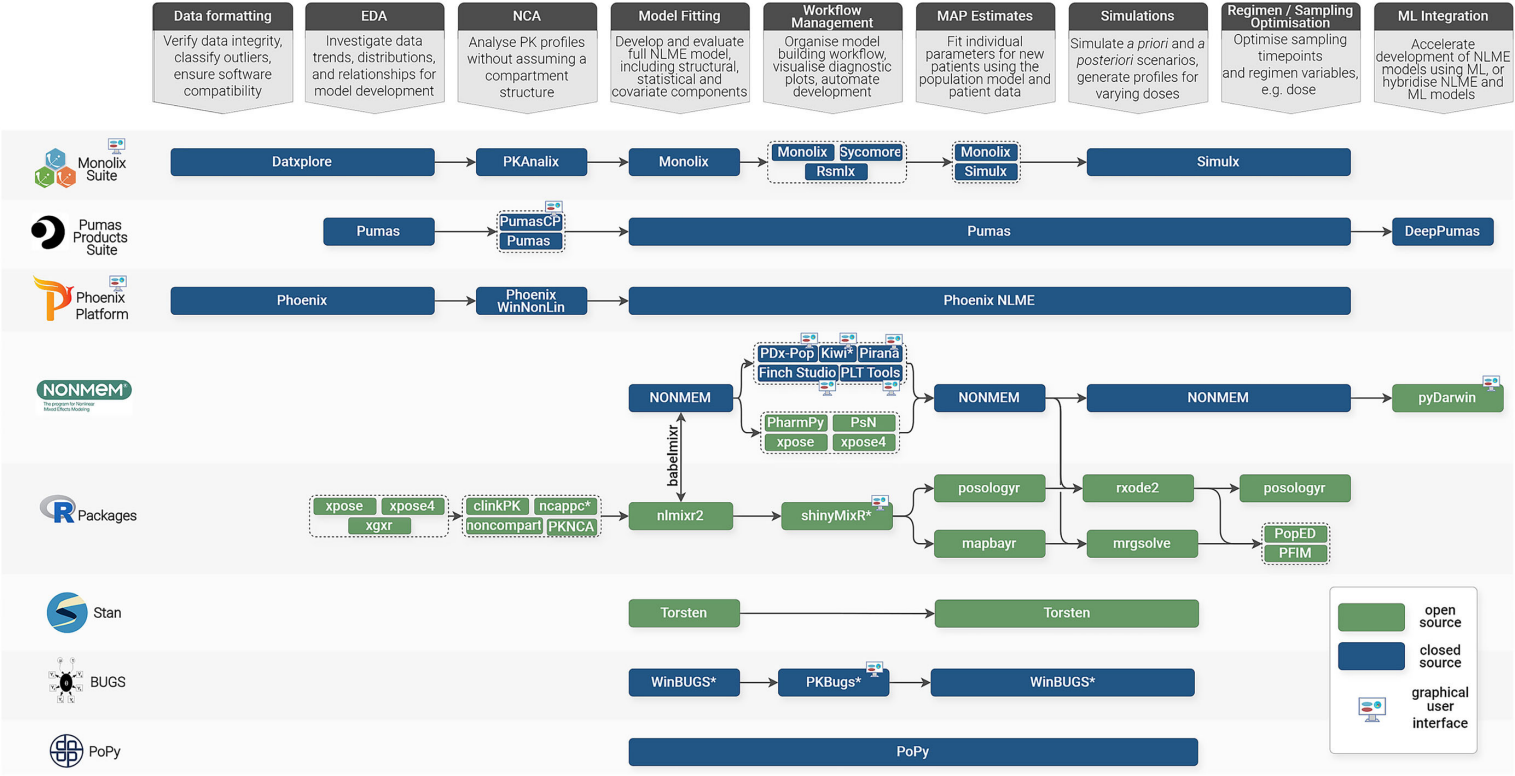
An illustrative map of population pharmacokinetics (popPK) modelling software packages across stages of the model building workflow. Tools marked with an asterisk (*) were last updated before 2020. EDA, exploratory data analysis; MAP, maximum *a posteriori*; ML, machine learning; NCA, compartmental analysis; NLME, nonlinear mixed‐effects.

Collectively, this presents a rich toolkit for popPK model development. However, it is important for the specific capabilities of the chosen software to align with the user's intended purpose and match their level of experience. For example, beginners might prefer graphical user interfaces (GUIs), which cover more of the modelling workflow and offer readily available diagnostic plots, (e.g. Monolix). Meanwhile, experienced users might favour the granular control and flexibility offered by combining tools such as NONMEM with open‐source R packages for visualisation and workflow management. Figure 2 maps key functionalities of some commonly used software across a typical PK modelling workflow to aid tool selection.

### 3.3 Nonlinear mixed‐effects model development

#### 3.3.1 Key steps in model development

After data handling and analysis planning, initial parameter estimates should be obtained (Figure 1A).^66^ Following this, the first component of the model to be determined is its structure (Figure 1B). Various structural models should be tested, starting with the simplest (i.e. smallest number of compartments) and increasing in complexity.^31^ Published models can also serve to inform the initial choice of an appropriate structural model. Covariates assumed to be influential on certain model parameters due to known physiological principles can be included in the structural model to improve stability in further model development.^31^

A statistical model, comprising residual unexplained variability (RUV), BSV and between‐occasion variability (BOV) where relevant, should then be added to the structural model to complete the base model (Figure 1C). Models for RUV should first be tested,^67^ which account for within‐subject variability and measurement error. Parameters accounting for random BSV and BOV (referred to as ETAs in NONMEM nomenclature) can be introduced in a stepwise fashion and retained if they provide significant improvement to relevant metrics and diagnostic plots.^68^ Correlations between ETAs and covariates, and correlations between ETAs themselves should also be assessed and accounted for.^68^

Following base model refinement, if there are potentially relevant covariates not yet included, a covariate model should be built (Figure 1D). The inclusion of covariates facilitates the stratification of patients based on predetermined characteristics, increases clinical interpretability and improves *a priori* model predictions^69^ (see Section 5.2). Several methods exist for covariate model building,^31^, ^69^, ^70^, ^71^, ^72^, ^73^, ^74^ with the most common being stepwise covariate modelling. Sanghavi *et al*.^69^ provide a detailed overview of covariate modelling approaches. The statistical model may be refined at this stage by removing some RUV, BSV or BOV parameters whose effects have been explained by covariates (Figure 1C).

The full covariate model should undergo a further stage of evaluation (Figure 1E) before it is accepted as the final model, including consultation of all model metrics and diagnostics. The final determined model should then be tested via sensitivity analysis, with the inclusion and exclusion of observations classified as outliers or previously excluded from the model building process.^31^, ^32^

#### 3.3.2 Metrics and diagnostics for basic internal validation

Several metrics can be used to evaluate the model during the building process.^68^, ^75^, ^76^ The most essential of these are the objective function value (OFV, measuring overall goodness‐of‐fit [GOF]), and the Akaike and Bayesian information criteria (measuring GOF while also penalising model complexity). The decrease in OFV from inclusion of most parameters can be tested for significance using the likelihood ratio test.^44^ While they give an indication of overall model fit, these metrics should not be considered in isolation; precedence should always be given to GOF plots and advanced diagnostics.

Alongside the above metrics, the following key GOF plots should be assessed at all stages of model development: observations *vs*. population predictions (PRED); observations *vs*. individual predictions (IPRED); conditional weighted residuals (CWRES) *vs*. time; CWRES *vs*. PRED; individual weighted residuals (IWRES) *vs*. time; IWRES *vs*. IPRED.^68^

#### 3.3.3 Metrics and diagnostics for advanced internal validation

The uncertainty of estimated parameters is another fundamental method of evaluating a model.^77^ Higher uncertainty indicates instability and a lack of reproducibility in parameter estimates. It will also lead to wider prediction intervals when forecasting future dosing scenarios (see Section 5), which reduces confidence in the model's predictive ability and hence undermines the clinical usability of dose recommendations. This can be quantified using parameter relative standard errors as well as correlations between parameters, which indicate redundancy or collinearity.^31^ Methods of estimating these values include using the inverse of the population Fisher Information Matrix (FIM)^68^, ^78^; bootstrapping^79^, ^80^; Sampling Importance Resampling (SIR)^81^ and log‐likelihood profiling.^77^ According to an analysis by Broeker and Wicha,^77^ the results of these methods differ marginally for large datasets. In the case of small datasets, the closest agreement with reference uncertainty distributions was attained by using SIR in conjunction with an initial proposal distribution generated using log‐likelihood profiling

The overall collinearity between parameters can also be assessed using the condition number of the covariance matrix.^44^ A model exhibiting severe collinearity (e.g. a condition number >1000^82^) should not be used for application in clinical practice due to instability in the estimated parameters.^31^

Various graphs (e.g. histogram or QQ plots) and statistical tests (e.g. Shapiro–Wilk, Jarque–Bera) can assess the normality of the distributions of ETAs, CWRES and IWRES.^44^, ^68^, ^83^ Since simulated values for statistical model parameters will typically be drawn from a normal distribution in most popPK software, it is important to validate the normality assumption to ensure simulation accuracy.

Finally, several simulation‐based diagnostics can assess how adequately the model represents the observed data, including visual predictive checks (VPCs)^79^, ^84^ and normalised prediction distribution errors.^85^, ^86^ These are effective, advanced tools in providing a preliminary *external* validation of the model, without the need for a separate dataset.^85^ However, they can become less interpretable as more variety is seen among patients and their treatment regimens (e.g. differing covariates, doses or sampling times).^87^ To allow the direct comparison across varied treatment regimens without the need for stratification (which can produce small, uninformative sample sizes^87^), prediction‐ and variance‐correction in VPCs can be used to normalise the observed and simulated concentrations values.^84^

### 3.4 Reporting

In response to the increasing use of popPK modelling, the European Medicines Agency published guidance in 2007 on reporting the results of popPK analyses to regulatory authorities.^88^ Whilst the recommendations were oriented towards a drug development perspective, learnings and merits of comprehensive reporting are transferable across the clinical spectrum. For example, complete popPK model reporting goes beyond ensuring reproducibility and comparability of study results,^89^ but also serves as guidance for regulatory evaluation whilst systematically outlining the novelty of the analysis compared to previous studies.^90^ Additionally, it facilitates the adaptation of literature models for use in MIPD software, as detailed in Section 4. The recommended structure of the final report has been described previously^52^, ^53^, ^89^, ^90^, ^91^ and is summarised in the Supplementary Information Section 3.

## 4 ADAPTATION OF LITERATURE MODELS

PopPK models for a wide range of drugs, populations and indications are available in open‐access journals.^92^ Procuring these models is an efficient strategy to scale MIPD software platforms as it reduces time needed to expand model libraries. Sourcing from reputable peer‐reviewed publications ensures the quality of a model has been preassessed, with contact to a corresponding author available should additional clarification be required.

Nonetheless, the use of pre‐established models also carries risk. Original models are often published within purely academic or research contexts with little consideration of their deployment into clinical practice. Therefore, the developer of MIPD software must bear responsibility for ensuring the validity of parameter estimations and dose recommendations based on those model outputs.^33^, ^34^ Hence, careful validation and verification is required to ensure suitability of a literature model for the target patient population (see Sections 5.1/5.3).

**FIGURE 3:**
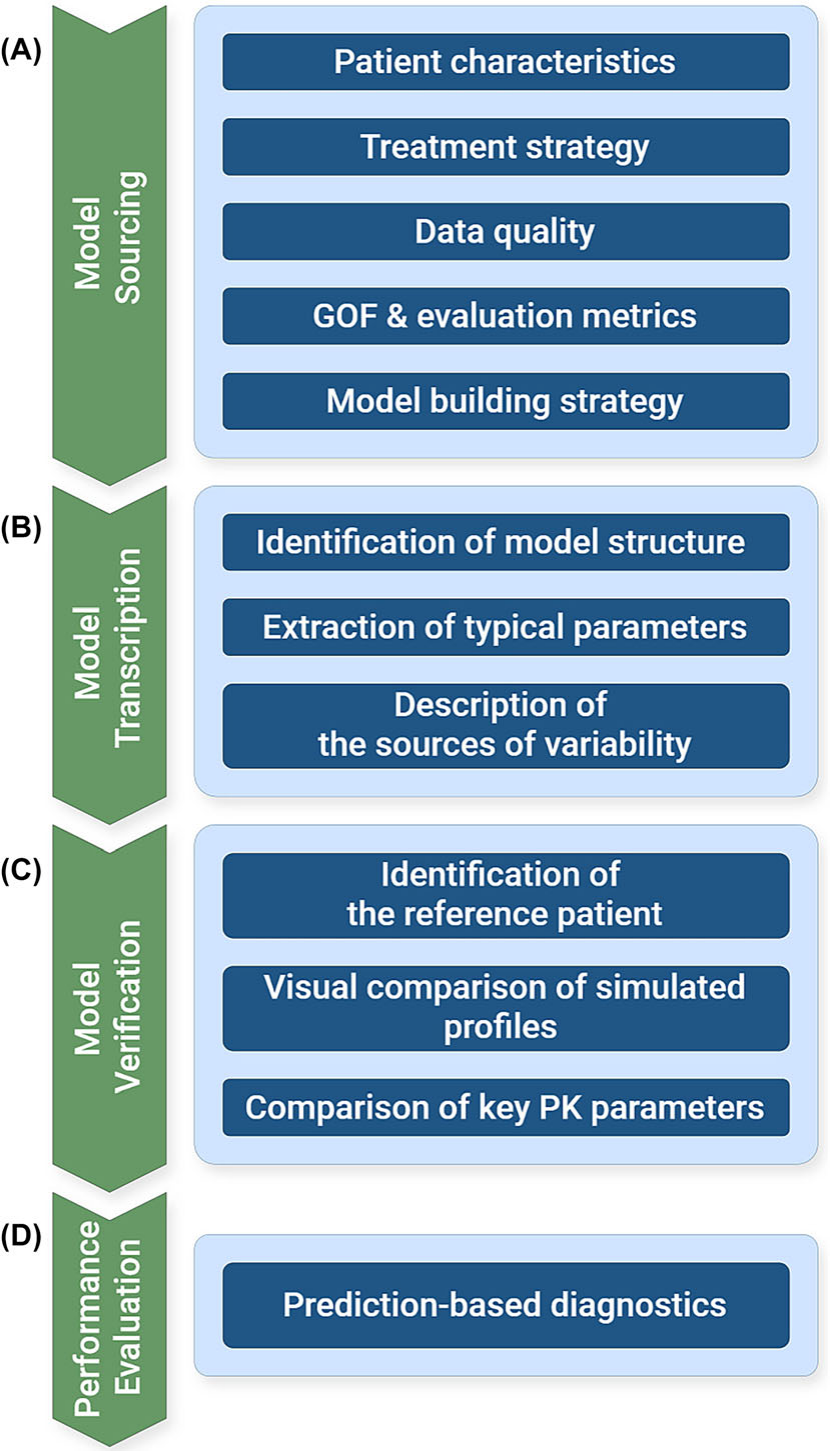
A workflow diagram summarising the process for adapting published population PK (popPK) models into model‐informed precision dosing software for their application in clinical practice, including (A) model sourcing, (B) model transcription, (C) model verification, (D) performance evaluation. GOF, goodness of fit; PK, pharmacokinetic. Adapted from Taylor *et al.* ^29^

Detailed guidance on selecting, adapting and validating literature models for inclusion in precision dosing software has been described previously.^29^ An overview is given below and is summarised in Figure 3.

### 4.1 Model sourcing

When sourcing candidate models from the literature (Figure 3A), consideration must be given to the patient population upon which the model was built. The demographics of the subjects included in model development should align with those of the target patient population to which MIPD will be applied.^87^ Similarly, the administration route and formulation, as well as sample collection (Section 2.1 and Supplementary Information Section 1.1) and processing protocols (e.g. sample matrix and sampling site) should agree between the model training population and the target population.^29^ There should be evidence that discrepancies in treatment regimen, comorbidities or concomitant therapies do not have a significant effect on drug disposition, as this would detract from the ability of the model to effectively describe the target population.^29^, ^93^ If the 2 populations are sufficiently aligned, the model's performance on the training population should be assessed using the same metrics and diagnostics described in Sections 3.3.6/3.3.7 and Supplementary Information Sections 2.3.6/2.3.7.

### 4.2 Model transcription

For a published model to be transcribed into an MIPD platform, all the relevant established parameters need to be identified and the model needs to specified according to the description in literature (Figure 3B).

As a minimum, replication of a popPK model requires:

- Structure of the model (e.g. 2‐compartment, can be inferred from the parameters listed);
- Typical population values for popPK parameters (e.g. for a 2‐compartment model: CL, V1, Q and V2);
- Covariate–PK parameter relationship equations, with final values of the covariate effect parameters specified (e.g. exponents in a power model);
- Parameters on which BSV and BOV were included, and the estimates for the variances of these distributions (often reported as CV%)^94^;
- Structure of residual error model (e.g. additive + proportional) and estimates for variances (also often reported as CV%);
- All units for the following: model parameters, doses and plasma concentrations.

Example workthroughs for the transcription of vancomycin models are provided in the Supplementary Information Section 4 for 2 distinct patient populations.

### 4.3 Model verification and evaluation

After the model is transcribed, its outputs need to be verified to ensure that this process has been done correctly. For that purpose, in order to allow for a fair comparison, a typical reference patient of the literature population should be identified. This is done based on the mean or median values of covariate factors as well as the associated ranges or distribution, which are typically reported along with a summary of patients' demographics in a popPK study.

Ideally, the study would also report graphical representations of model outputs that can be used to comparatively assess the successful transcription of the model (Figure 3C). Some examples include:

- VPCs, to compare with simulations ran on patients created based on typical covariates;
- Example plasma concentration profiles to indicate a range of concentrations that can be expected for a typical patient;
- Simulated plasma concentration profiles, ideally for a specified patient or for a typical patient.

If none of the above examples are available, the strategy can be adapted to reflect the level of information provided. For example, specific PK parameters such as AUC, *C*_*max*_ (the highest observed concentration value) or *C*_24_ (the concentration value after 24 hours) may be reported where full profiles are not provided. In such scenarios, successful transcription of the model can be determined by calculating and comparing the same metric.

Before using the adapted model at point of care, its performance should be evaluated on external validation data (Figure 3D), as described in Section 5.3.

## 5 MODEL APPLICATION IN HEALTHCARE

**FIGURE 4:**
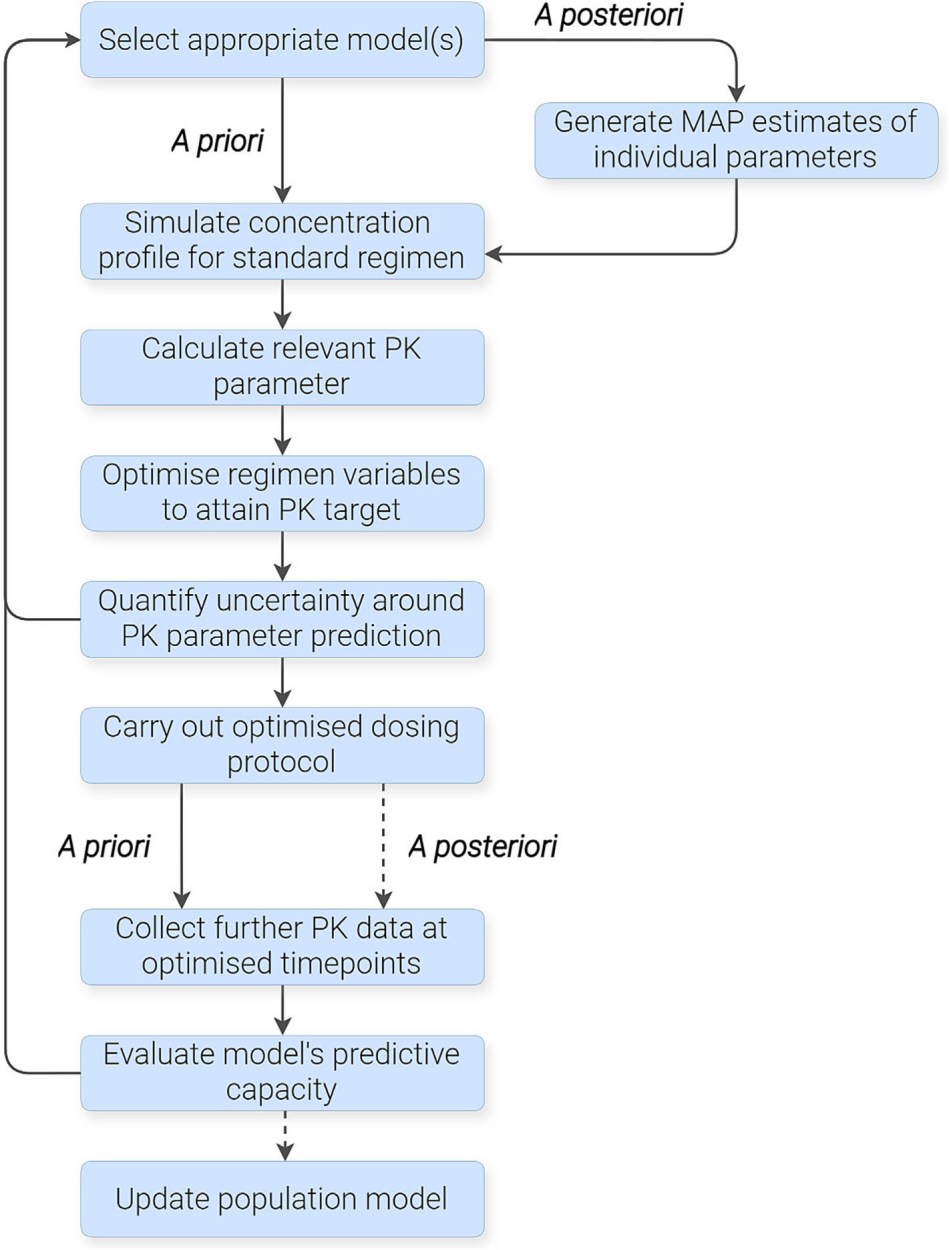
Workflow diagram illustrating the application of population PK models to optimise treatment. Dashed arrows indicate steps to be performed after several iterations of the preceding workflow have been carried out. MAP, maximum *a posteriori*; PK, pharmacokinetic.

Whether a model has been built directly from raw data or adapted from the literature into MIPD software, further steps remain to determine how best to use it at point of care, as described in the following subsections and summarised in Figure 4.

### 5.1 Model selection

Where several models for a drug are available within an MIPD platform, the most appropriate 1 to describe the PK of a given patient needs to be determined. This can be done initially by comparing relevant patient covariates and any other factors related to their condition with the available models. If the patient's covariate values lie at the extremities (or outside) of the ranges seen in the model's training population, flattened priors can be used when fitting individual parameter estimates to any of the patient's available PK data.^87^ Here, the influence of population model parameter value priors is downweighed in favour of information from the PK data. This leads to a risk of overfitting to the data which can cause inaccurate parameter estimates. Further PK data should always be collected to validate parameter estimates based on flattened priors.^87^

Success has also been seen through using multiple models (developed in heterogeneous populations and indications) and automatically selecting the best‐performing or averaging their results.^95^ The OFV or Akaike information criteria of the models' fit to patient PK samples can be used to rank models for selection, or determine their weight in the averaging process.^95^ This allows the use of collective findings of previous modelling studies and automatically and objectively handles the burden of choosing the model that is most applicable to a given patient. In doing so, it reduces risk of misattribution and increases robustness against misspecification of individual models. This is particularly beneficial for implementing dose recommendations in a clinical setting and improves usability of the MIPD software.

### 5.2 A priori *vs.* a posteriori predictions

Model predictions can be classified as *a priori*, where only the patient's covariates are available to the model, or *a posteriori*/*posthoc*, where some of the patient's drug concentration data have been used to fit the model to the individual.^15^, ^96^, ^97^ In the former scenario, the patient's PK parameters are set to the typical values expected for a patient with the given covariates (determined by the covariate–parameter relationships specified in the model), i.e. the prediction is a *PRED* value. Meanwhile, *a posteriori* estimates use both covariates and individual ETA values (maximum *a posteriori* [MAP] estimates) obtained when fitting for the individual, and hence give a personalised *IPRED* value for prediction.^96^ Predicting future dosing scenarios using MAP estimates and updated values for any time‐varying covariates is referred to as Bayesian forecasting.^15^, ^97^

Where an appropriate model is available, *a priori* predictions allow the starting dose to be optimised to reach the desired PK target and can be beneficial for those without the on‐site capacity to quantify drug levels.^15^ Most commercial MIPD software platforms offer such an approach^27^; however, *a priori* predictions may result in imprecise estimates and have come under scrutiny in the literature.^15^, ^22^, ^98^, ^99^ This is particularly the case when patient covariate characteristics fall outside of the validated range, which limits the model's extrapolation properties and its potential to be applied to new patients in a clinical setting. It has been shown that including even a single timed sample can provide marked improvements over *a priori* estimates, with the addition of further samples increasing their precision.^15^, ^99^ In 1 case, inclusion of PK measurements for vancomycin resulted in a 135‐fold reduction in bias of PK parameter estimates.^97^ In the case of fludarabine, it has been highlighted that an overreliance on pharmacometric models that only use covariate data can increase the risk of imprecise estimates.^21^, ^22^, ^100^ Therefore, where individual patient's drug levels can be obtained, *a posteriori* dose prediction should be favoured over *a priori* predictions.

### 5.3 Model evaluation and uncertainty quantification

Before a model is used for dosing decisions at point of care, it is crucial to evaluate its predictive capacity. There is currently no set standard on how to verify popPK model credibility; however, the American Society of Mechanical Engineers Verification and Validation 40^101^, ^102^ provides a framework for model verification and validation that is increasingly adopted as gold‐standard in model‐informed drug development contexts,^103^ suggesting a similar template for MIPD in clinical practice. The lack of clarity around the need and method for model evaluation has led to inconsistent approaches applied in published models,^76^ with some analyses showing that only 28% of popPK models underwent advanced internal validation (e.g. bootstrapping, VPCs etc.) and <7% were externally validated on test data.^104^

Moreover, there is no widespread agreement on what metrics and acceptability thresholds should be considered when evaluating model predictions on test data.^105^ For instance, accuracy and precision can be commonly measured via mean prediction error and root mean square error respectively. However, the percentage thresholds of root mean square error or mean prediction error deemed *acceptable* must be considered on a case‐by‐case basis, with their impact on the desired clinical outcome as the determining factor. For example, a more stringent acceptability threshold should be applied for a drug with a narrower therapeutic window.^105^

The generation of confidence and prediction intervals is important to quantify uncertainty around the model's prediction. This can be done in several ways for a, NLME model,^106^ including by:

1. Approximation using the model's Jacobian matrix and the parameter uncertainty matrix (i.e. the inverse of the individual's FIM);
2. Repeatedly resimulating the prediction after sampling new parameter values from the model's parameter uncertainty distributions and variability distributions (for BSV, BOV and RUV);
3. Using the predictions of several alternate models obtained by repeatedly re‐estimating the model parameters on generated data (either simulated by the model or bootstrapped from the original dataset).

Despite being computationally inexpensive, the approximation required in method 1 can lead to inaccuracies, particularly for very nonlinear models. While method 3 has been shown to perform best, and requires no underlying assumptions on the uncertainty distributions, it has large computational costs. Also, parameters obtained by re‐estimation on bootstrapped data may be overly sensitive to outliers, unless a large dataset is available. For these reasons, method 2 is preferred as a compromise to avoid approximation inaccuracies, excessive computational complexity and the need for large datasets.^106^

For *a priori* predictions for an individual, the parameter uncertainty matrix can be calculated using the individual's FIM, allowing the implementation of any of the methods described by Kümmel *et al*.^106^ Similarly, the Bayesian FIM can be used to estimate this matrix for *a posteriori* prediction, where MAP estimates are used for individual parameters.^107^, ^108^ Maier *et al*.^107^ explored several other methods for quantifying uncertainty around MAP estimates for an individual and found sequential Bayesian data assimilation to be the most computationally efficient compared with using the FIM, SIR or Monte Carlo Markov Chain methods.^107^ Uncertainty around MAP estimates and its resulting effect on prediction uncertainty can be used to guide the need for PK sample collection, since a larger number of samples will typically result in a reduction in uncertainty.^87^

Using methods 2 or 3, for example, the PK parameter of interest (e.g. AUC, *C*_*max*_) can be calculated for each resimulated plasma concentration curve. If the targeted value is defined by a range (e.g. therapeutic window), then the percentage of simulations lying within, above and below this range can be calculated. If instead a single value is targeted (e.g. as in TCI), then the arithmetic mean of the samples can be used as the expected value for the PK target, and relevant percentiles used to create a (e.g. 95%) confidence interval around it.^106^ This information can be displayed to the user of the MIPD software to assess the likelihood of safe and efficacious treatment, and hence assist them in deciding whether to accept the dosing recommendation.

### 5.4 Treatment and sampling optimisation

In both the *a priori* and *a posteriori* cases, alternative dosing simulations can predict the expected values of relevant PK targets.^109^ These simulations allow for the adjustment of the administered dose to achieve the desired PK results and optimise patient outcomes. For example, AUC can be calculated via numerical integration of the simulated PK curve, or in simple scenarios (e.g. for a 1‐compartment infusion model), through analytical expressions that consider the patient's clearance and target AUC value.

If the target is the average steady‐state concentration, the dose can be calculated according to Equation 1:

(1) $$D=\frac{\mathrm{CL}\cdot C_{\mathrm{avg}}}{\tau }$$

where *D* = dose; *CL* = clearance rate; *C*_avg_ = average target concentration; *τ* = dosing interval.

As a general solution, an optimisation algorithm (e.g. gradient descent) can be used to identify the correct dose required to obtain the desired PK target.^110^ This approach can also be applied to optimise other aspects of the treatment scenario, for example infusion duration or dosing intervals to adjust *C*_*max*_, though this is uncommon.

For cases where the target is a therapeutic window, the centre of the window can be selected as the initial target, and uncertainty quantification can be applied to assess the likelihood of maintaining concentrations within this window. If the confidence intervals around predictions are asymmetric, an alternative point within the window may be targeted to minimise the risk of under‐ or overexposure. This flexibility ensures that individual patient variability is accounted for during dosing optimisation.^7^

The workflow for regimen optimisation up to this point can be carried out using historic patient data (i.e. data that precede the use of MIPD software) if any are available. Any data collected after MIPD software is available should be collected at optimal timepoints as determined by the PK model.^36^ These timepoints (or windows) can also be ascertained through D‐optimality using the FIM,^36^, ^37^, ^108^, ^111^, ^112^ with the objective of capturing the greatest amount of PK information in the most efficient manner. This can be done at population level using the population FIM, for *a priori* optimisation, while the Bayesian FIM can be used to determine optimal timepoints for an individual *a posteriori*.^108^

Optimising sample collection time is essential for the application of MIPD for TDM at point of care, since the collection of TDM data is labour intensive, intrusive and burdensome for the patient. In doing so, more effective predictions can be obtained with fewer PK samples, ensuring the impact on clinical workflows can be minimised. As mentioned in Section 2.1, it may be practically infeasible to collect samples at the analytically determined optimal timepoints. One approach to mitigate this is to sample at the viable time closest to the theoretically determined optimal timepoint. Optimal sampling windows can also be calculated, as opposed to targeting fixed time points, to allow more flexibility for clinical implementation.^112^

For drugs where the trough concentration is targeted, this cannot be calculated as simply as the steady‐state approach. Trough targets require advanced modelling techniques to predict the concentration at the end of the dosing interval. However, recent studies have shown that trough concentrations may not always be the best proxy for drug exposure. For example, in the case of vancomycin, Wicha *et al*. demonstrated that AUC is a better predictor of both efficacy and safety, and trough levels may be misleading in terms of therapeutic outcomes.^15^

### 5.5 Continuous integration of patient data

Over time, a patient's physiology, and hence their reaction to a given drug, can be expected to change. Part of this change can be attributed to time‐varying covariates (e.g. weight or renal function), while the remaining unexplained variability between occasions can be quantified using ETA parameters for BOV.

When PK data are available from only 1 dosing event, but the relevant model contains both BSV and BOV parameters, BSV and BOV cannot be distinguished. Hence MAP estimates for BSV and BOV cannot be simultaneously calculated; BOV must be ignored and BSV estimated alone.^87^ In this scenario, it is important to collect PK samples from multiple dosing events in order to accurately distinguish the effects of BSV from BOV and improve the accuracy of MAP estimates for the individual. This facilitates more realistic simulations of future dosing scenarios, allowing for more accurate determination of an optimal dose in a clinical setting.^87^

Once PK data are available from multiple historical occasions, MAP estimates for both BSV and BOV can be determined, but further considerations are required for how they should be generated. Guo *et al*.^113^ explored several methods, including using data from all occasions with equal weighting; weighting the historical data chronologically; and iteratively updating the MAP estimates based on data from each occasion sequentially. The highest accuracy and precision were observed by using the final method.^113^

Different implementation strategies are available for how to use MAP estimates for Bayesian forecasting when both BSV and BOV are included in the model, as described by Abrantes *et al*.^114^ The best performing approach was found to include BOV in the generation of the MAP estimates but exclude the portion of variability related to BOV from the individual parameters used in Bayesian forecasting.

Just as PK parameter estimates for an individual must be adapted over time, continuous learning can be used to improve population models as more data are collected.^115^, ^116^ This can be done using a sequential hierarchical Bayesian framework and is particularly beneficial for model generalisability (especially for PK models built on limited data), although challenges remain when new data for inclusion are sparsely sampled.^116^ It should also be noted that the updating and deployment of modified population models has regulatory implications with respect to when newer versions can be made available for clinical use.^34^

## 6 SOFTWARE INTEGRATION WITH CLINICAL PRACTICE

### 6.1 Integration with digital healthcare infrastructure

Pharmacological models require fit‐for‐purpose software platforms for effective clinical deployment. A core challenge is establishing an integrated data pipeline with the dosing platform.^27^ It is common for individual healthcare records to be fragmented across multiple databases and systems. However, principal healthcare organisations typically hold MIPD‐relevant patient information on centralised electronic health record (EHR) systems, such as Epic,^117^ Oracle Health^118^ or MEDITECH.^119^

Digitisation of health records makes the interoperability with EHRs an essential requirement to widely deploy precision dosing tools and integrate seamlessly with clinical workflows.^28^ A variety of integration pathways exist, which affect the development process, software architecture, deployment complexity, functional characteristics and user adoption.

Traditionally, custom software integration involved building these tools into pre‐existing EHR technology stacks at each primary treatment organisation. This meets the usability requirements easily, but scales poorly due to lack of generalisability.^120^ Moreover, it consumes significant time and resources from the developer and exposes the healthcare provider to risk in terms of security and flexibility. Therefore, software developers are increasingly adopting scalable off‐the‐shelf systems from EHR vendors.

The first scalable vendor integration pathway^121^, ^122^ handles data transfer via an application programming interface but gives access to the user via an independent interface, e.g. as a web application. Whilst increasing the complexity of deployment, this offers an independence advantage by leaving the responsibility for data access and support with the EHR vendor. However, complexity scales with the number of sought vendor compatibilities, requiring multiple data access and authentication methods.

An alternative approach involves publishing the dosing tool as an application within the vendor's EHR platform,^121^, ^122^ broadening accessibility and facilitating distribution. However, this places constraints upon the interface development, requiring compliance with vendor protocols. In this context, the data access methods follow either the vendor application programming interface approach, or the standardised Fast Healthcare Interoperability Resources (FHIR) approach.

The Health Level Seven International^123^ organisation has published frameworks and American National Standards Institute‐accredited standards, covering the exchange, integration, sharing and retrieval of electronic health information. Healthcare providers can expose FHIR‐compliant servers to third parties seeking access to patient data, ensuring standardised data annotation and transparent requests.^123^ FHIR does not mandate a single technical approach but provides a set of building blocks that can be applied to create secure, private systems. Thus, compliance with frameworks such as FHIR is critical for MIPD interoperability across EHR databases.

### 6.2 Regulation and quality control

MIPD software tools used in clinical settings may be classified as medical devices or clinical decision support software, depending on their intended application and jurisdiction of use.^27^, ^33^, ^124^ Software as a medical device requires quality and regulatory compliance with specifications published by international standards organisations like the International Electrotechnical Commission (IEC) or the International Organisation for Standardisation (ISO). This is monitored during development, upon submission to the regulatory authorities and maintained during the lifecycle of use.

Software as a medical device development requirements include implementation of a quality management system that adheres to ISO 13485^34^ and IEC 62304,^125^ both of which ensure that user needs are met and that product specifications are validated throughout the software lifecycle. Risks need to be constantly evaluated and mitigated in accordance with ISO 14971,^126^ which also addresses usability engineering considerations as per IEC 62366.^127^

Given the sensitivity of input data, cybersecurity is a significant consideration. This is addressed by software compliance with information security management systems standards (e.g. ISO/IEC 27001)^128^ ^,^ ^129^, ^130^, ^131^, ^132^, ^133^, ^134^, ^135^ alongside mandatory General Data Protection Regulation and/or the Health Insurance Portability and Accountability Act legislation.

## 7 OUTLOOK FOR MIPD

Proponents of MIPD are advancing beyond the deployment of standard NLME models to enhance clinical outcomes. Leading this progression is the application of machine learning to expedite and optimise model building and dose recommendations.

Several recent reviews provide detail on the opportunities and pitfalls of applying ML into popPK modelling and MIPD.^136^, ^137^, ^138^, ^139^, ^140^, ^141^, ^142^ These include the capacity of ML algorithms to discern hidden patterns in data more easily,^137^ while also presenting challenges around result interpretability.^140^ Several proof‐of‐concept works have successfully demonstrated replacing popPK models with ML models for PK parameter prediction,^143^ hybridising NLME and ML models to improve upon MAP parameter estimates,^115^ using ML algorithms to accelerate NLME model development^144^ and creating more realistic synthetic PK data by use of ML.^145^

However, use of ML in dose recommendations carries specific regulatory implications.^146^, ^147^, ^148^, ^149^ For example, in March 2024 the EU adopted the Artificial Intelligence (AI) Act which classifies the use of AI in healthcare as *high‐risk* and is thus subject to multiple compliance requirements.^150^

As the field of AI continues to grow and interoperability with EHRs improves, opportunities for AI integration with MIPD will expand.^136^ However, it is imperative that MIPD software developers continue to align with best practice for ML development^151^ and AI policy^150^, ^152^ to ensure patient safety and facilitate trust.

## 8 CONCLUSION

The current MIPD landscape is evolving, driven by a common goal to individualise patient treatment and optimise health outcomes. Despite substantial progress, several barriers hinder the widespread adoption of MIPD in clinical practice. These include the complexity of implementing popPK models as a precision dosing aid, variability in clinical workflows, different cost‐to‐benefit ratios between drugs and inconsistency in adopted methods. Additionally, healthcare providers take time to adapt to new technologies and integrate them into existing clinical and reimbursement systems.

To overcome these challenges, a multifaceted consideration of best practice is necessary. This involves developing standardised guidelines that cover the entire precision dosing workflow, including data handling, model development, validation and application, as outlined in this manuscript. Further steps will include enhancing interoperability with EHRs through frameworks such as FHIR, and facilitating communication between stakeholders, including regulatory bodies, healthcare providers and MIPD software developers. This perspective highlights the current issues and establishes clearer pathways for the adoption of precision dosing tools. This can ensure more consistent and reliable dose optimisation, and ultimately lead to a higher standard of patient care.

## AUTHOR CONTRIBUTIONS

M.B. and I.H. led the manuscript preparation and primary drafting, with substantial input from A.Z. and A.B. J.S. and A.T. contributed to the conceptualisation of the manuscript and provided editing support. A.T. and M.P. contributed to early drafts. S.B., G.V. and K.O. reviewed and provided critical feedback on the manuscript.

## CONFLICT OF INTEREST STATEMENT

Monika Berezowska, Isaac S Hayden, Arsenii Zats, Mehzabin Patel, Alaric Taylor and Jugal Suthar are employees of Vesynta Ltd. Alaric Taylor and Jugal Suthar are shareholders in Vesynta Ltd. Gareth Veal serves as a board advisor to Vesynta Ltd. Kayode Ogungbenro is a paid consultant of Vesynta Ltd. The remaining authors have no conflicts of interest to declare.

## Supporting information


**DATA S1** Supporting Information.

**DATA S2** Supporting Information.

**DATA S3** Supporting Information.

**DATA S4** Supporting Information.

## Data Availability

Data sharing is not applicable to this article as no new data were created or analyzed in this study.
