# Supplementary material for: Recommended approaches for integration of population pharmacokinetic modelling with precision dosing in clinical practice
Source: Br J Clin Pharmacol. 2024 Nov 21;91(4):1064–79. doi: 10.1111/bcp.16335 (PMC11992666; doi:10.1111/bcp.16335)
Supplement: Supplementary file 1 — DATA S1 Supporting Information. [file BCP-91-1064-s001.pdf]

SUPPLEMENTARY MATERIALS

Recommended Approaches for Integration of Population Pharmacokinetic Modelling with Precision Dosing in Clinical Practice

Short title: Integration of population pharmacokinetic models with precision dosing

Monika Berezowska[1†] | Isaac S Hayden[1†] | Andrew M Brandon[2] | Arsenii Zats[1] | Mehzabin Patel[1] | Shelby Barnett[2] | Kayode Ogungbenro[3] | Gareth J Veal[2] | Alaric Taylor[1] | Jugal Suthar[1]

[1]Vesynta Ltd, Innovation Gateway, The London Cancer Hub, Cotswold Road, Sutton, London, UK

[2]Translational and Clinical Research Institute, Newcastle University Centre for Cancer, Newcastle upon Tyne, UK

[3]Division of Pharmacy & Optometry, School of Health Sciences, University of Manchester, Manchester, UK

Correspondence
Dr Jugal Suthar, Vesynta Ltd, Innovation Gateway, The London Cancer Hub, Cotswold Road, Sutton, London, UK
Email: jugal.suthar@vesynta.com

Other Emails:
monika.berezowska@vesynta.com
isaac.hayden@vesynta.com
andrew.brandon@newcastle.ac.uk
arsenii.zats@vesynta.com
mehzabin.patel@vesynta.com
shelby.barnett@newcastle.ac.uk
kayode.ogungbenro@manchester.ac.uk
gareth.veal@newcastle.ac.uk
alaric.taylor@vesynta.com

Funding information
This publication is an output from a wider research initiative financially supported by the UKRI (Project ID 10111236)

Current methods of dose determination have contributed to sub-optimal and inequitable health outcomes in underserved patient populations. The persistent demand to individualise patient treatment, alongside increasing technological feasibility, is leading to a growing adoption of model-informed precision dosing (MIPD) at the point of care. Population pharmacokinetic (popPK) modelling is a technique that supports treatment personalisation by characterising drug exposure in diverse patient groups. This publication addresses this important shift in clinical approach, by collating and summarising recommendations from literature. It seeks to provide standardised guidelines on best practices for the development of popPK models and their use in MIPD software tools, ensuring the safeguarding and optimisation of patient outcomes. Moreover, it consolidates guidance from key regulatory and advisory bodies on MIPD software deployment, as well as technical requirements for electronic health record integration. It also considers the future application and clinical impact of machine learning algorithms in popPK and MIPD. Ultimately, this publication aims to facilitate the incorporation of high-quality precision dosing solutions into standard clinical workflows, thereby enhancing the effectiveness of individualised dose selection at the point of care.

KEYWORDS
pharmacometrics, therapeutic drug monitoring, translational research, modelling and simulation, prescribing

[†]MB and IH contributed equally to this work

1 | DATA CONSIDERATIONS FOR MODEL DEVELOPMENT

Pharmacokinetic (PK) data used for model building may originate from multiple sources, including clinical trials with differing protocols. Crucially, model development may not be a core objective behind the PK data generation [1]. Thus, the data may be limited or inconsistent in terms of formatting, available patient information, accuracy of sampling times, or reported measurement units [2, 3, 4]. When prospectively planning studies, PK-informed trial design can support the suitability of data generated for model building [5, 6, 7], as discussed below. The following sections also provide a brief discussion of approaches to data formatting and common strategies for handling missing or erroneous data.

1.1 | Data collection

Establishing a data pipeline with standardisation of collection and processing ensures regulatory compliance [8, 9], reproducibility, traceability and accuracy - all critical factors for effective application in model-informed precision dosing (MIPD).

Where the PK of a drug has been studied in humans, the optimum number and timings of samples can be influenced by the PK parameter of interest or can be determined using statistical methods described in Main Text Section 5.4 [6, 7, 10]. In practice, optimal sample timepoints may be unfeasible due to logistical constraints. Opportunistic or scavenged sampling regimens may need to be adopted instead to reduce the burden on patients, especially in, e.g., neonatal and paediatric populations [5].

When applied to patients from these and other under-represented populations, population pharmacokinetic (popPK) models built on a relatively homogenous, widely studied patient cohort are frequently found to translate poorly [11]. Though methods exist to mitigate this (see Main Text Section 5.1), the U.S. Food & Drug Administration (FDA) has drafted guidance expressing the importance of collecting data from specific and diverse patient sub-populations. This supports the building of robust models that accurately represents the patient group and, by extension, ensures equitable access to healthcare [12].

1.2 | Formatting data

In 2020, the International Society of Pharmacometrics (ISoP) set out a recommended data structure for use in popPK modelling and noncompartmental analysis (NCA) [13]. The formatting of data for use in PK modelling largely depends on the software that will be used for model development. Previous publications have described software-specific data formatting requirements [14, 15, 16]. In 2018, the PharmaSUG Software Users Group published a short paper [17] on data standards for popPK and NCA, which included a discussion on data cleaning and formatting.

Generally, data labels should be short but easily identifiable, and logically ordered for both the user and the software [13]. Consideration should be given to whether data are continuous or categorical variables. The latter should be flagged with appropriate numerical values, with a clear record kept for interpretability. From an industry perspective, to create a clear, traceable, and reproducible record, data formatting should be carried out using unit tested methods and version-controlled software or scripts (as opposed to manual data manipulation) to process raw data files, which are retained in original form [18, 19].

1.3 | Data exploration and cleaning

Early visual inspection and statistical analysis can help identify patterns and potential data outliers, as well as informing the structural and statistical models [3, 20]. Diagnostic tools, including statistical summaries and correlation matrices, can give an overview of covariate distributions and relationships [3, 20]. More fundamentally, this process may identify unexpected, erroneous or missing data [4]. The approach chosen for handling such data [3, 13] can have significant impact on the accuracy and precision of final model estimates [3, 4].

Any missing or abnormal data in case report forms should be investigated by consulting associated documents, clinical research staff and laboratory scientists as appropriate [4]. If this data is not recoverable, the patient may have to be removed from the dataset. A preferred alternative can be to calculate dose timings or to estimate covariate values from context, where possible [4, 13]. However, care should be taken to ensure the chosen method of imputation is appropriate [4], and that the values used are traceable.

It is also common for sample concentrations to be below the lower limit of quantification (BLLOQ) of the bioanalytical assay used. Numerous methods are available for handling such data [3, 21, 22, 23, 24, 25], with the most appropriate choice being influenced by the proportion of patient samples that is BLLOQ, the specific structural PK model, and how rich or sparse the remaining data are [4, 21]. If these points constitute <10% of the modelling dataset, then excluding them has been shown to have little effect on the accuracy of estimated model parameters [3, 4]. Alternatively, other methods can be applied to account for BLLOQ data to avoid a systemic bias where a model underestimates drug clearance [23].

The choice of inclusion method for BLLOQ data can also be influenced by consideration of computation time and probability of successful convergence. To improve the accuracy of estimated parameters regardless of previously mentioned constraints, the implementations that perform best are the M3 and M4 methods, which maximise the likelihood of flagged data points being BLLOQ [4, 21, 22, 23]. Alternatively, setting all BLLOQ points to LOQ/2 shortens run times and improves the probability of successful convergence during model development, however, doing so may introduce considerable bias to the model [21, 23]. An effective strategy can be to initially include BLLOQ data as LOQ/2 during model building and utilise the M3 or M4 method towards the end of the model development process. The model may then require some minor refinement, but this minimises the risk of non-convergence, speeds up development, and reduces computational costs while still retaining parameter accuracy in the final model.

Regardless of decisions made during the data cleaning process, all processing actions should be recorded with justification, for inclusion in the model report to support validity assessment (see Supplementary Information Sections 2.4 and 3).

2 | MODEL BUILDING

In 2022, the FDA published updated guidance on popPK analysis for new drug applications [10], with recommendations ranging from data collection and analysis to model building and reporting. Other prominent publications have described this process from a more technical perspective [14, 3, 26, 27]. Additionally, the pharmacometrics community share useful tools and knowledge about model development [28, 29, 30, 31, 32, 33]. The recommended process of building a popPK model described in these resources is consolidated below and summarised in Figure 1.

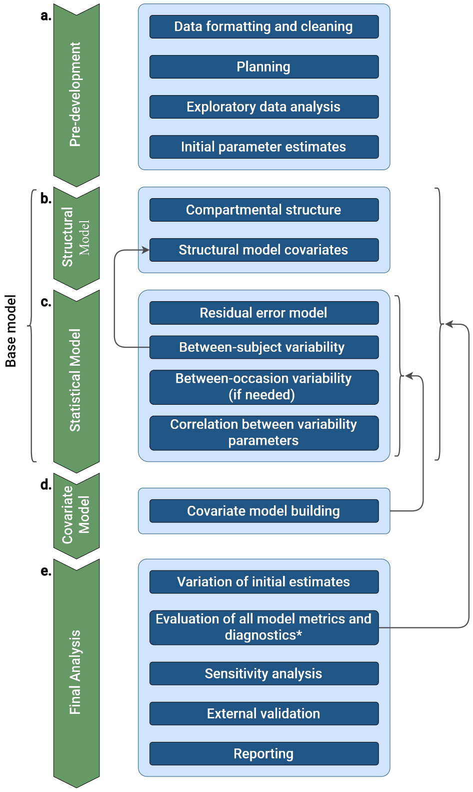

FIGURE 1 A workflow diagram of the recommended steps in the pharmacokinetic model development process: **a.** Pre-development, **b.** Structural model, **c.** Statistical model, **d.** Covariate model, **e.** Final analysis. * Appropriate metrics and diagnostics should be considered throughout the building process (see Supplementary Information Sections 2.3.6/2.3.7), but all should be considered collectively at this step. Adapted from Byon et al. [3].

2.1 | Planning model development

Prior to popPK model development, it is best practice to prepare a population modelling analysis plan (PMAP) (Figure 1a) [34, 35]. The PMAP should clearly state the purpose and objectives of analysis and briefly describe the data-originating study (if applicable), followed by a review from experienced modellers to ensure appropriateness and quality of the modelling strategy [35].

Planning should specify the modelling methodology, data handling procedures, information available prior to the study, and any assumptions made by the modeller [3, 34]. In addition, it should propose structural and variability models to be tested, with a rationale based on biological, pharmacological, or clinical plausibility [34]. This includes provisions that establish criteria for model selection and covariate inclusion (e.g., statistical significance or other metrics as detailed in Supplementary Information Section 2.3) [36, 37]. The robustness and predictive performance of the model can be ensured by including procedures for external model validation (see Main Text Section 5.3) [34]. The choice of model building software and parameter estimation algorithms to be used should also be justified.

2.2 | Software

The earliest software for popPK model building was NONMEM [38]; hence many available guidelines and publications are oriented around NONMEM nomenclature. Since then, numerous licensed software tools, such as Monolix, Pumas, Phoenix NLME and PoPy [39, 40, 41, 42] have been developed and adopted. Moreover, open-source R packages such as nlmixr2, posologyr, mapbayr, mrgsolve and rxode2 [43, 44, 45, 46, 47, 48, 49] have gained popularity, which either replicate, complement or augment the functionality of the privately licensed software. Generic statistical modelling packages like Stan and BUGS have also seen use in publications [50, 51].

Collectively, this presents a rich toolkit for popPK model development. However, it is important for the specific capabilities of the chosen software to align with the user's intended purpose and match their level of experience. For example, beginners might prefer code-free interfaces which cover more of the modelling workflow and offer readily available diagnostic plots (e.g., Monolix). Meanwhile, experienced users might favour the granular control and flexibility offered by combining tools like NONMEM with open-source R packages for visualisation and workflow management. Figure 2 maps key functionalities of some commonly used software across a typical PK modelling workflow to aid tool selection.

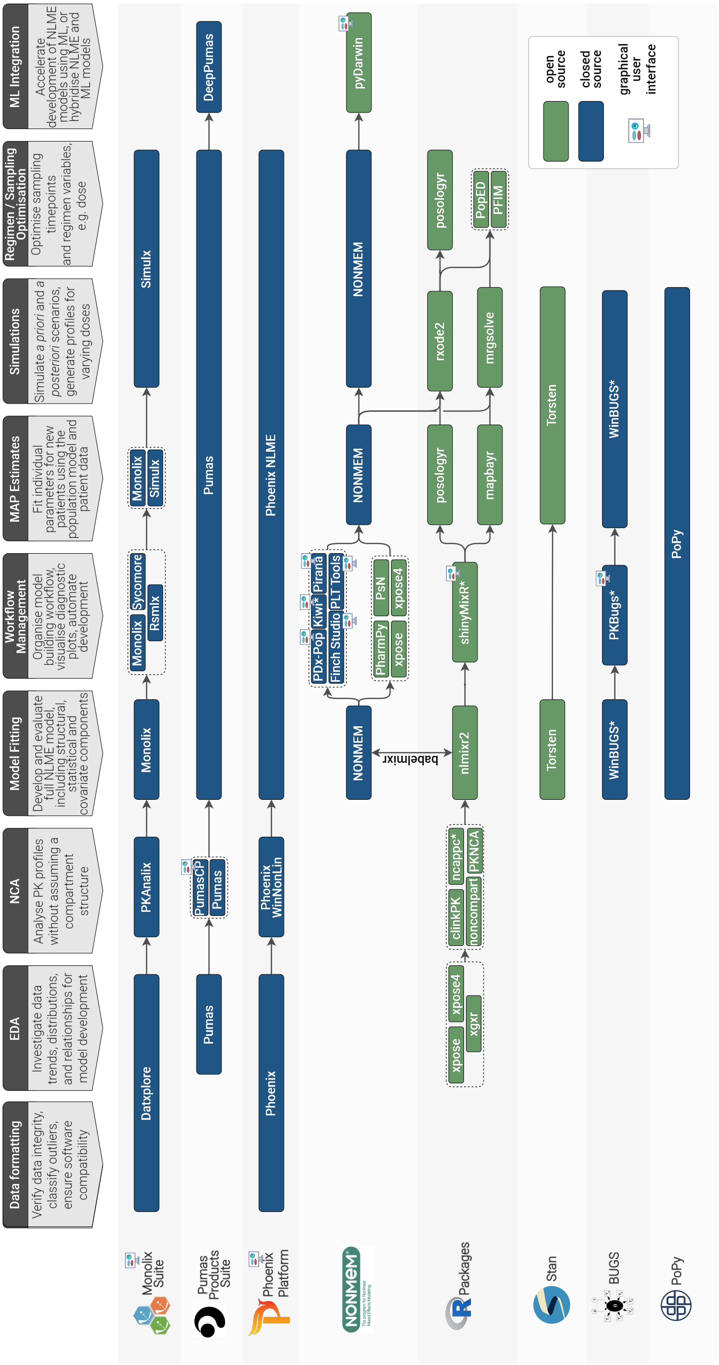

FIGURE 2 An illustrative map of population pharmacokinetics (popPK) modelling software packages across stages of the model building workflow. Tools marked with an asterisk (*) were last updated before 2020. EDA: Exploratory data analysis, NCA: Non-compartmental analysis, NLME: Nonlinear mixed-effects, MAP: Maximum *a posteriori*, ML: Machine learning.

2.2.1 | Estimation algorithms

Development packages host several estimation algorithms for fitting model parameters to observed data. Traditional linearisation methods (e.g., first-order conditional estimation (FOCE)) are deterministic and computationally inexpensive but rely on approximation, which can reduce accuracy [27]. Methods utilising random sampling (e.g., stochastic approximation expectation maximisation (SAEM)) are being increasingly adopted due to their robustness with sparse datasets. Data collected for population PK studies is often sparse, and hence these methods may be preferred for point-of-care applications. However, these have larger computational costs and can have lower precision due to stochastic variability [27]. Differences in performance of various algorithms have been explored extensively in several reviews [27, 52, 53, 54, 55].

2.3 | Nonlinear mixed-effects model development

2.3.1 | Pre-development

After data handling and analysis planning, initial parameter estimates should be obtained (Figure 1a). Appropriate initial values can assist in the successful convergence of parameter estimation algorithms, and can be sourced from literature, or estimated using analyses such as NCA, naive averaged data, naive pooled data, or standard two-stage approaches [56, 57].

2.3.2 | Structural model

The first component of the model to be determined is its structure (Figure 1b). Various structural models should be tested, starting with the simplest (i.e., smallest number of compartments) and increasing in complexity [3]. Published models can also serve to inform the initial choice of an appropriate structural model. Covariates assumed to be influential on certain model parameters due to known physiological principles can be included in the structural model to improve stability in further model development [3].

2.3.3 | Statistical model

A statistical model, comprising residual unexplained variability (RUV), between-subject variability (BSV), and between-occasion variability (BOV) where relevant, should then be added to the structural model to complete the base model (Figure 1c). Models for RUV should first be tested [58], which account for within-subject variability and measurement error. A common approach is to initially include both an additive and a proportional term for residual error (combined error model), with the proportional component partly relating to bioanalytical assay response and the additive component relating to instrument noise [58]. One of these terms may then become unnecessary (as indicated by a reduction in magnitude) as variability is sufficiently explained by other parameters later during model development [26]. If log-transformed data are being modelled (this is commonly done to add stability, particularly if concentration values span multiple orders of magnitude), then careful consideration should be given to how different error models are interpreted. For example, a proportional error model on a linear scale becomes additive on a log scale (see Mould and Upton (2013) [26] for more examples).

Parameters accounting for random BSV and BOV are referred to as ETAs in NONMEM nomenclature. These can be introduced in a stepwise fashion and retained if they provide significant improvement to relevant metrics and diagnostic plots as described in Supplementary Information Sections 2.3.6/2.3.7 [59]. Correlations between ETAs and

covariates can be assessed to inform the inclusion of other influential covariates in the base model [59], which may in turn remove the need for some ETAs as variability becomes explained. Correlations between ETAs themselves should then be examined and accounted for in the model if significant. This facilitates realistic PK parameter combinations to accurately simulate variability in future dosing scenarios when optimising patient treatment [3].

2.3.4 | Covariate model

Following base model refinement, if there are potentially relevant covariates not yet included, a covariate model should be built (Figure 1d). The inclusion of covariates facilitates the stratification of patients based on predetermined characteristics, increases clinical interpretability and improves *a priori* model predictions [60] (see Main Text Section 5.2). Several methods exist for covariate model building [3, 60, 61, 62, 63, 64, 65], with the most common being stepwise covariate modelling (SCM). SCM involves the systematic forward inclusion of all considered covariates, one at a time, where the most influential covariate effect (based on predefined acceptance criteria) is retained at each stage, and the process repeated with remaining covariates until no further significant improvement is observed [63]. Covariates are then removed in a similar manner (backward elimination), but with stricter retention criteria, until only the most influential covariates remain [63]. While this is a stable algorithm and saves manually testing covariate inclusions, SCM is computationally intensive and, as a greedy algorithm, it may converge to a local minimum and hence miss important combinations of covariate effects [60]. Furthermore, it only considers statistical significance of covariate effects and is unable to assess other key metrics and diagnostics (see Supplementary Information Sections 2.3.6/2.3.7 below) [60].

Many alternative approaches to covariate modelling exist, see Sanghavi et al. [60] for a detailed overview. Two recent developments include Stochastic Approximation for Model Building Algorithm (SAMBA) and Conditional Sampling for Stepwise Approach based on Correlation tests (COSSAC).

SAMBA [64] allows for faster and less computationally intensive screening of covariates than SCM. This is achieved by simultaneously introducing the most influential covariates to multiple parameters during each iteration, thus reducing the total number of combinations tested. This allows the modeller to quickly ascertain key covariate-parameter relationships but may not always identify the optimal combination [64].

Similarly, COSSAC [65] guides parameter-covariate relationship testing by analysing the correlations between covariates and ETAs. These methods may be beneficial when the model structure is more complex, or when many covariates need to be tested, in which cases SCM may fail to converge within a reasonable time. A balance can be achieved by using SAMBA or COSSAC to provide rapid preliminary insights in selecting covariate information for inclusion in a full SCM analysis [65].

The statistical model may be refined at this stage by removing some RUV, BSV or BOV parameters whose effects have been explained by covariates (Figure 1c).

2.3.5 | Final analysis

The full covariate model should undergo a further stage of evaluation (Figure 1e) before it is accepted as the final model. The range of initial parameter estimates previously collected should be tested (and randomly perturbed) to reduce the risk of convergence to local minima during optimisation [3]. At this point, all model metrics and diagnostics should be consulted again, as detailed in Supplementary Information Sections 2.3.6/2.3.7. This evaluation may provide evidence for removing certain covariate effects or statistical model parameters. Alternatively, it may indicate significant model misspecification, in which case the structural model should be revisited, and all subsequent steps repeated. The final

determined model should then be tested via sensitivity analysis, with the inclusion and exclusion of observations classified as outliers or previously excluded from the model building process [3, 4].

2.3.6 | Metrics and diagnostics for basic internal validation

Several metrics can be used to evaluate the model during the building process [59, 66, 67]. The most essential of these are the objective function value (OFV, measuring overall goodness-of-fit (GOF)), and the Akaike and Bayesian Information Criteria (AIC and BIC, respectively, measuring GOF while also penalising model complexity). The decrease in OFV from inclusion of most parameters can be tested for significance using the likelihood ratio test (LRT) [26]. When including statistical model parameters (for BSV, BOV and RUV), the underlying statistical assumptions of the LRT are not satisfied, and so the AIC and BIC should be consulted instead [26]. While they give an indication of overall model fit, these metrics should not be considered in isolation; precedence should always be given to GOF plots and advanced diagnostics discussed below. Two other key metrics are ETA-shrinkage, which measures the redundancy of ETA parameters, and EPSILON-shrinkage, which indicates overfitting of individual predictions (IPRED)) [26, 68, 69]. Studies have shown that high shrinkage (more than 20-30%) increases parameter estimation bias and renders GOF plots uninformative and misleading [68].

Alongside the above metrics, the following key GOF plots should be assessed at all stages of model development: observations vs population predictions (PRED); observations vs IPRED; CWRES vs time; CWRES vs PRED; individual weighted residuals (IWRES) vs time; IWRES vs IPRED [59]. Care should be taken when interpreting these plots, as even a well-specified model may not exhibit the “expected pattern” for each graph (e.g., the observations vs PRED plot should be evenly distributed around the line of identity) [70]. The pattern seen in the observed data should instead be compared to reference plots created using simulated data from the candidate model and dosing regimen of interest. More detail on this method is provided by Karlsson and Savic [70].

2.3.7 | Metrics and diagnostics for advanced internal validation

The uncertainty of estimated parameters is another fundamental method of evaluating a model [71]. Higher uncertainty indicates instability and a lack of reproducibility in parameter estimates. It will also lead to wider prediction intervals when forecasting future dosing scenarios (see Main Text Section 5), which reduces confidence in the model’s predictive ability and hence undermines the clinical usability of dose recommendations. This can be quantified using parameter relative standard errors (RSEs) as well as correlations between parameters, which indicate redundancy or collinearity [3]. The most common method of estimating these values utilises the inverse of the population Fisher Information Matrix (FIM) [59, 72]. While this is a fast and easy method, it relies on the assumption of normally distributed uncertainty distributions, and numerical issues often make the inverse of the FIM difficult to compute [71]. Bootstrapping [73, 74] is a common approach to avoid these issues but comes with large computational cost and is sensitive to population outliers [71]. More recent methods using Sampling Importance Resampling (SIR) [75] and Log-Likelihood Profiling (LLP) are gaining popularity as less computationally intensive alternatives to bootstrapping [71]. According to an analysis by Broeker and Wicha [71], the results of these methods differ marginally for large datasets. In the case of small datasets, the closest agreement with reference uncertainty distributions was attained by using SIR in conjunction with an initial proposal distribution generated using LLP.

The overall collinearity between parameters can also be assessed using the condition number of the covariance matrix [26]. Although there is no exact threshold for what constitutes an acceptable condition number, a typical interpretation is that a condition number >1000 is considered severe collinearity [76]. In the presence of sparse data,

collinearity may be unavoidable and may be accepted in academic contexts. However, a model exhibiting severe collinearity should not be used for application in clinical practice due to instability in the estimated parameters [3].

Various graphs (e.g., histogram or QQ plots), and statistical tests (e.g. Shapiro-Wilk, Jarque-Bera) can assess the normality of the distributions of ETAs, CWRES and IWRES [26, 59, 77]. Since simulated values for statistical model parameters will typically be drawn from a normal distribution in most popPK software, it is important to validate the normality assumption to ensure simulation accuracy. Where significant non-normality is observed, this can be addressed by transformations [78, 79], although these can lead to overfitting when used on small datasets. To avoid the need for transformations, some software now allows the use of non-normal distributions for statistical model parameters [39, 40].

Finally, several simulation-based diagnostics can assess how adequately the model represents the observed data, including visual predictive checks (VPCs) [73, 80] and normalised prediction distribution errors (NPDEs) [81, 82]. These are effective, advanced tools in providing a preliminary "external" validation of the model, without the need for a separate dataset [81]. However, they can become less interpretable as more variety is seen among patients and their treatment regimens (e.g., differing covariates, doses or sampling times) [11]. A common solution involves stratifying patients based on key variables; however, this can render samples sizes too small for the graphs to be informative [11]. To allow the direct comparison across varied treatment regimens without the need for stratification, prediction- and variance-correction in VPCs can be used to normalise the observed and simulated concentrations values [80].

2.3.8 | Automatic model development

Since the development of a popPK model is a complex, multi-phase process, new automated model building tools have emerged in recent years, with the aim of streamlining the model development process [83, 84]. Within these development tools, a multi-dimensional search space can be specified, which can include characteristics such as the number of compartments, the statistical model structure, covariates to be considered and the covariate-parameter relationships. Typically, these tools use algorithms which revolve around stepwise inclusion and/or backwards elimination of features [64, 85, 84], sharing many of the same limitations as the SCM process (Supplementary Information Section 2.3.4).

More recently, there has been increasing interest in replacing these with machine learning (ML)-based approaches (see Main Text Section 7) [86]. A recent study compared the efficiency of several methods in identifying the optimal combination of model features, benchmarked against an exhaustive search [86]. While all methods were able to find the best model in an improved computation time, Gaussian processes were the most efficient.

Although automatic approaches have great potential to facilitate model development with reduced human input, the outputs of these methods still need to be verified by the modeller to ensure their utility in clinical practice [87]. Specifics of the algorithms used, such as hyperparameters and model evaluation criteria, also need to be customised to ensure that the automatic model development process converges successfully to the optimal model.

2.4 | Reporting

In response to the increasing use of popPK modelling, the European Medicines Agency (EMA) published guidance in 2007 on reporting the results of popPK analyses to regulatory authorities [88]. Whilst the recommendations were oriented towards a drug development perspective, learnings and merits of comprehensive reporting are transferable across the clinical spectrum. For example, complete popPK model reporting goes beyond ensuring reproducibility and comparability of study results [89], but also serves as guidance for regulatory evaluation whilst systematically

outlining the novelty of the analysis compared to previous studies [90]. Additionally, it facilitates the adaptation of literature models for use in MIPD software, as detailed in Main Text Section 4.

To attain these benefits, a report should explain the purpose of the analysis, assess the adequacy of the final model and discuss implications of the results on clinical practice. The PMAP can be included as an appendix to the report. However, the plan must be written prior to the study, whereas the report should be continuously maintained as a live document as the study progresses. Any deviations from the originally proposed strategy and methods in the PMAP should be reported with a clear explanation of the change [34].

The recommended structure of the final report has been described previously [34, 35, 89, 90, 91] and is summarised in Supplementary Information Section 3, below.

3 | POPULATION PHARMACOKINETIC MODELLING REPORT STRUCTURE

A frequently recommended structure of a final population pharmacokinetic modelling building report is as follows [34, 35, 89, 90, 91].

Synopsis: A brief outline of the objectives and impact of the analysis, summarising data, methodology and key findings with the supporting evidence. The adequacy of the data can be visually justified for evaluating exposure differences in specific populations. The results can be presented in terms of drug exposure metrics (e.g. AUC, C_{min}, C_{max} and C_{avg}) to illustrate covariate impacts on pharmacokinetics.

Introduction: Provide background information and context on the pharmacokinetic characteristics of the drug. Clearly state the primary and secondary objectives, explain the motivation for the analysis, summarise the available analysis, and highlight the research gap the study aims to fill.

Data: Describe the origins of data, study design, data handling procedures and any modifications to the original dataset. The concentration profiles can be plotted here to show the patterns in the raw data and briefly discuss the number of patient and points per patients. Detail the data used, including assay characteristics and procedures for handling missing data and outliers.

Methods: Detail the software, fitting algorithms, estimation methods, and criteria for covariate inclusion. Describe the model structure, including structural, variability, and covariate models, and present sensitivity analyses.

Results: Summarise data exploration with graphs and tables, describe model development and evaluation, and present diagnostics and validation. Use tables and figures to illustrate simulation results and model applications, such as the impact of significant covariates on PK parameters.

Discussion: Interpret the results in a physiologic and mechanistic context without merely repeating them. Explain their clinical relevance, discuss the impact of covariates on pharmacokinetics, and assess the robustness of findings considering assumptions and limitations. Evaluate alternative dosing regimens where relevant.

Conclusion: Provide a succinct summary of the major findings and their impact, written in nontechnical language. This should be a single paragraph or a bullet list.

Appendix: Include the original analysis plan, supplemental tables and figures, a list of omitted data points with reasons, model code and outputs, and a run record of the analysis steps. Provide methods and code for generating key figures, ensuring compliance with the FDA preferences for code submission.

4 | MODEL TRANSCRIPTION: WORKED EXAMPLES

Two vancomycin popPK models [92, 93], representing two distinct patient populations (adults undergoing allogeneic hematopoietic stem-cell transplantation [92], and paediatrics with febrile neutropenia [93]) were selected to provide worked examples of model transcription, as discussed in Main Text Section 4.2.

Following identification of a published target model, both key and otherwise contextual information is extracted, and an in-house template (Supplementary Material 2) is populated. The extracted information is then used to build, in this case, a NONMEM control stream file which can then be used to run simulations for verification and comparison to a reference dataset, where available.

4.1 | Example 1: Adults

A model reported by Okada et al. [92] describing the popPK of vancomycin in adult patients undergoing allogeneic hematopoietic stem-cell transplantation was used for this model transcription.

First, an in-house template was populated using the contents of the published article. The filled in template can be found in the Supplementary Material 3. Collecting information beyond the minimum requirement for model transcription ensures comprehensive recording of the source study, model features, and information on the population on which the model was built, including patient characteristics beyond those included in the model itself. Publications reporting popPK models may sometimes omit information, and this is noted in the template as 'Not specified'.

Any relevant figures or graphs would then be saved alongside the completed template, for comparison with any simulations.

Information gathered on parameter estimates, model structure and covariate inclusion was then used to recreate the model as a NONMEM control stream. As the parameter estimates provided will not be estimated by the software (as a $ESTIMATION or $EST block is not included), parameter values may or may not be fixed in the code.

For example:

```
$PROBLEM vancomycin_modeltranscription1
; Model transcription of Okada et al. 2018, J Clin Pharmacol 58(9)
;---------------------------------
$INPUT ID AMT RATE TIME DV SEX AGE WT BSA CLCR EVID MDV OUTL
;---------------------------------
$DATA dataset-for-validating.csv IGNORE=@
;---------------------------------
$SUBROUTINES ADVAN3 TRANS4
;---------------------------------
$PK
TVCL   = THETA(1)
TVV1   = THETA(2)
TVQ    = THETA(3)
TVV2   = THETA(4)

CLCRCL = (CLCR/113)**THETA(5)
WTV    = (WT/59.4)**THETA(6)
```

```
IIVCL  = EXP(ETA(1))
IIVV1  = EXP(ETA(2))
IIVV2  = EXP(ETA(3))

CL   = TVCL * CLCRCL * IIVCL
V1   = TVV1 * WTV * IIVV1
Q    = TVQ
V2   = TVV2 * IIVV2
S1   = V1/1000 ; scale concentration units
;-----------------------------------
$THETA
4.25 FIX  ; (1) CL
39.2 FIX  ; (2) V1
1.95 FIX  ; (3) Q
56.1 FIX  ; (4) V2
0.70 FIX  ; (5) CLCRCL
0.78 FIX  ; (6) WTV
;----------------------------------
$OMEGA
0.063   ; IIV CL (ETA1)  |  IIVs from paper back-transformed with n=(%CV/100)^2
0.0202  ; IIV V1 (ETA2)
0.4476  ; IIV V2 (ETA3)
;----------------------------------
$SIGMA
0.172  ; ERROR
;----------------------------------
$ERROR
Y = F * (1+EPS(1))
;----------------------------------
$SIM ONLYSIM (54321) SUBPROBLEMS=1000
;----------------------------------
$TABLE ID TIME DV MDV ONEHEADER NOPRINT NOAPPEND IDFORMAT=I FILE=vancsim001.tab
```

4.2 | Example 2: Paediatrics

A model reported by Shimamoto et al. [93] describing the popPK of vancomycin in paediatric patients with febrile neutropenia was used for this model transcription.

As discussed above, the in-house template was first populated with information from the published article (Supplementary Material 4).

Any relevant figures or graphs would then be saved alongside the completed template, for comparison with any simulations.

Information gathered on parameter estimates, model structure and covariate inclusion was then used to recreate the model as a NONMEM control stream.

For example:

```
$PROBLEM vancomycin_modeltranscription2
; Model transcription of Shimamoto et al. 2021, J Antimicrob Chemother 76
;----------------------------------
$INPUT ID AMT RATE TIME DV PMA WT SCR GFR BT EVID MDV OUTL
;----------------------------------
$DATA dataset-for-validating.csv IGNORE=@
;----------------------------------
$SUBROUTINES ADVAN3 TRANS4
;----------------------------------
$PK
TVCL  = THETA(1)
TVV1  = THETA(2)
TVQ   = THETA(3)
TVV2  = THETA(4)

WTCLQ = (WT/70)**THETA(5)
WTV   = (WT/70)**THETA(6)
EMAX  = PMA**THETA(8)/(THETA(7)**THETA(8) + PMA**THETA(8))
GFRCL = (GFR/120)**THETA(9)

IF(BT>=38) THEN
FLAG = 1
ELSE FLAG = 0
ENDIF ; if BT is >= 38C, then BTCL (THETA(10)) is included
BTCL  = THETA(10)**FLAG

IIVCL  = EXP(ETA(1))

CL   = TVCL * WTCLQ * EMAX * GFRCL * BTCL * IIVCL
V1   = TVV1 * WTV
Q    = TVQ * WTCLQ
V2   = TVV2 * WTV
S1   = V1/1000 ; scale concentration units
;-----------------------------------
$THETA
5.94 FIX  ; (1) CL
39.9 FIX  ; (2) V1
3.85 FIX  ; (3) Q
37.8 FIX  ; (4) V2
```

```
0.75 FIX  ; (5) WTCLQ
1.0 FIX   ; (6) WTV
47.7 FIX  ; (7) TM50
3.4 FIX   ; (8) HILL COEFFICIENT
0.626 FIX ; (9) GFRCL
1.12 FIX  ; (10) BTCL
;-----------------------------------
$OMEGA
0.052   ; IIV CL (ETA1)
;-----------------------------------
$SIGMA
0.0855  ; ERROR
;-----------------------------------
$ERROR
Y = F * (1+EPS(1))
;-----------------------------------
$SIM ONLYSIM (54321) SUBPROBLEMS=1000
;-----------------------------------
$TABLE ID TIME DV MDV ONEHEADER NOPRINT NOAPPEND IDFORMAT=I FILE=vancsim002.tab
```

References

[1] Taylor ZL, Poweleit EA, Paice K, Somers KM, Pavia K, Vinks AA, Punt N, Mizuno T, Girdwood ST. Tutorial on model selection and validation of model input into precision dosing software for model-informed precision dosing. CPT: Pharmacometrics & Systems Pharmacology, 2023;12:1827–1845.

[2] Mould D, Upton R. Basic Concepts in Population Modeling, Simulation, and Model-Based Drug Development. CPT: Pharmacometrics & Systems Pharmacology, 2012;1:1–14.

[3] Byon W, Smith MK, Chan P, Tortorici MA, Riley S, Dai H, Dong J, Ruiz-Garcia A, Sweeney K, Cronenberger C. Establishing best practices and guidance in population modeling: an experience with an internal population pharmacokinetic analysis guidance. CPT: Pharmacometrics & systems pharmacology, 2013;2:51.

[4] Irby DJ, Ibrahim ME, Dauki AM, Badawi MA, Illamola SM, Chen M, Wang Y, Liu X, Phelps MA, Mould DR. Approaches to handling missing or "problematic" pharmacology data: Pharmacokinetics. CPT: Pharmacometrics and Systems Pharmacology, 2021;10:291–308.

[5] Laughon MM, Benjamin DK, Capparelli EV, Kearns GL, Berezny K, Paul IM, Wade K, Barrett J, Smith PB, Cohen-Wolkowiez M. Innovative clinical trial design for pediatric therapeutics. Expert Review of Clinical Pharmacology, 2011;4:643–652.

[6] Aarons L, Ogungbenro K. Optimal Design of Pharmacokinetic Studies. Basic & Clinical Pharmacology & Toxicology, 2010;106:250–255.

[7] Chenel M, Ogungbenro K, Duval V, Laveille C, Jochemsen R, Aarons L. Optimal Blood Sampling Time Windows for Parameter Estimation Using a Population Approach: Design of a Phase II Clinical Trial. Journal of Pharmacokinetics and Pharmacodynamics, 2005;32:737–756.

[8] Regulation (EU) 2017/745 of the European Parliament and of the Council of 5 April 2017 on medical devices, amending Directive 2001/83/EC, Regulation (EC) No 178/2002 and Regulation (EC) No 1223/2009 and repealing Council Directives 90/385/EEC and 93/42/EEC; 2017. https://eur-lex.europa.eu/legal-content/EN/TXT/?uri=CELEX%3A02017R0745-20240709.

[9] British Standards Institution. Medical devices - Quality management systems - Requirements for regulatory purposes (ISO 13485); 2016. https://www.evs.ee/en/evs-en-iso-13485-2016.

[10] The United States Food & Drug Administration. Population Pharmacokinetics: Guidance for Industry; 2022. https://www.fda.gov/regulatory-information/search-fda-guidance-documents/population-pharmacokinetics.

[11] Dorlo TP, Svensson EM. Pharmacokinetic–Pharmacodynamic Modeling and Simulation in Clinical Practice and Studies. In: Advances in Pharmacokinetics and Pharmacodynamics Springer; 2023.p. 109–135.

[12] U.S. Food and Drug Administration. Diversity Action Plans to Improve Enrollment of Participants from Underrepresented Populations in Clinical Studies Guidance for Industry; 2024. https://www.fda.gov/regulatory-information/search-fda-guidance-documents/diversity-action-plans-improve-enrollment-participants-underrepresented-populations-clinical-studies.

[13] International Society of Pharmacometrics Data Standards: Basic data structure for population pharmacokinetic (popPK) analysis; 2020.

[14] Bauer RJ. NONMEM Tutorial Part I: Description of Commands and Options, With Simple Examples of Population Analysis. CPT: Pharmacometrics & systems pharmacology, 2019;8:525–537.

[15] Simulations Plus Monolix 2024R1 User Guide; 2024. https://monolix.lixoft.com/single-page/.

[16] Fidler M, Wilkins JJ, Hooijmaijers R, Post TM, Schoemaker R, Trame MN, Xiong Y, Wang W. Nonlinear Mixed-Effects Model Development and Simulation Using nlmixr and Related R Open-Source Packages. CPT: Pharmacometrics & systems pharmacology, 2019;8:621–633.

[17] Thanneer N. Data standards for population pharmacokinetic analysis (POPPK) and non-compartmental analysis (NCA). In: PharmaSUG 2018 - Paper AA-16 Bristol-Myers Squibb Company; 2018. .

[18] Adane K, Gizachew M, Kendie S, The role of medical data in efficient patient care delivery: A review. Dove Medical Press Ltd; 2019.

[19] Dash S, Shakyawar SK, Sharma M, Kaushik S. Big data in healthcare: management, analysis and future prospects. Journal of Big Data, 2019;6.

[20] Sun H, Fadiran EO, Jones CD, Lesko L, Huang SM, Higgins K, Hu C, Machado S, Maldonado S, Williams R, Hossain M, Ette EI. Population pharmacokinetics. A regulatory perspective. Clinical pharmacokinetics, 1999;37:41–58.

[21] Beal SL. Ways to fit a PK model with some data below the quantification limit. Journal of pharmacokinetics and pharmacodynamics, 2001;28:481–504.

[22] Ahn JE, Karlsson MO, Dunne A, Ludden TM. Likelihood based approaches to handling data below the quantification limit using NONMEM VI. Journal of Pharmacokinetics and Pharmacodynamics, 2008;35:401–421.

[23] Bergstrand M, Karlsson MO. Handling Data Below the Limit of Quantification in Mixed Effect Models. The AAPS Journal, 2009;11:371–380.

[24] Byon W, Fletcher CV, Brundage RC. Impact of censoring data below an arbitrary quantification limit on structural model misspecification. Journal of Pharmacokinetics and Pharmacodynamics, 2008;35:101–116.

[25] Duval V, Karlsson MO. Impact of omission or replacement of data below the limit of quantification on parameter estimates in a two-compartment model. Pharmaceutical Research, 2002;19:1835–1840.

[26] Mould D, Upton R. Basic Concepts in Population Modeling, Simulation, and Model-Based Drug Development—Part 2: Introduction to Pharmacokinetic Modeling Methods. CPT: Pharmacometrics & Systems Pharmacology, 2013;2:1–14.

[27] Bauer R. NONMEM Tutorial Part II: Estimation Methods and Advanced Examples. CPT: Pharmacometrics & systems pharmacology, 2019;8:538–556.

[28] Lavielle M, WikiPopix Mixed effects models for the population approach; 2013. https://wiki.inria.fr/popix/Home_page.

[29] Navaro D, Notes from a Data Witch; 2024. https://blog.djnavarro.net/.

[30] NM Users Mail Archive; 2024. https://www.mail-archive.com/nmusers@globomaxnm.com/.

[31] Sassen SDT, Agema BC, PK-PD Info; 2023. https://pkpd-info.com/index.php.

[32] Bourne D, PharmPK; 2023. https://www.pharmpk.com/.

[33] Guo T, Hyper-NM75help; 2024. https://nmhelp.tingjieguo.com/index.htm.

[34] Wade JR, Edholm M, Salmonson T. A guide for reporting the results of population pharmacokinetic analyses: a Swedish perspective. The AAPS journal, 2005;7:45.

[35] Bonate PL, Strougo A, Desai A, Roy M, Yassen A, van der Walt JS, Kaibara A, Tannenbaum S. Guidelines for the quality control of population pharmacokinetic-pharmacodynamic analyses: an industry perspective. The AAPS journal, 2012;14:749–758.

[36] Bonate PL. Recommended reading in population pharmacokinetic pharmacodynamics. The AAPS journal, 2005;7:E363–373.

[37] Barker CIS, Standing JF, Kelly LE, Faught LH, Needham AC, Rieder MJ, de Wildt SN, Offringa M. Pharmacokinetic studies in children: recommendations for practice and research. Archives of disease in childhood, 2018;103:695–702.

[38] Beal SL, Sheiner LB, Boeckmann AJ, Bauer RJ, NONMEM 7.5. Icon Development Solutions; 2023.

[39] SAS L, Monolix 2023R1. Lixoft; 2023.

[40] Pumas-AI, Pumas: Pharmaceutical Modeling and Simulation. Pumas-AI; 2023.

[41] Certara, Phoenix NLME 8.3. Certara; 2023.

[42] Wright Dose Ltd, PoPy: Population Pharmacokinetics in Python; 2024.

[43] Fidler M. nlmixr2: Nonlinear Mixed Effects Models in Population PK/PD; 2024, https://nlmixr2.org/.

[44] Leven C, Coste A, Mané C. Free and Open-Source Posologyr Software for Bayesian Dose Individualization: An Extensive Validation on Simulated Data. Pharmaceutics, 2022;14(2):442.

[45] Easy and reliable maximum a posteriori Bayesian estimation of pharmacokinetic parameters with the open-source R package mapbayr. CPT: Pharmacometrics & systems pharmacology, 2021;10:1208–1220.

[46] Baron KT. mrgsolve: Simulate from ODE-Based Models; 2024, https://github.com/metrumresearchgroup/mrgsolve, r package version 1.5.1.

[47] Fidler ML, Hallow M, Wilkins J, Wang W. RxODE: Facilities for Simulating from ODE-Based Models; 2024, https://CRAN.R-project.org/package=RxODE, r package version 2.1.3.

[48] Norris DC, noncompart: Non-compartmental Analysis for Pharmacokinetic Data. R Foundation for Statistical Computing; 2023.

[49] Acharya C, Hooker AC, Türkyılmaz GY, Jönsson S, Karlsson MO. A diagnostic tool for population models using non-compartmental analysis: The ncappc package for R. Computer methods and programs in biomedicine, 2016;127:83–93.

[50] Stan Development Team, Stan: A C++ Library for Probability and Sampling; 2024. https://mc-stan.org.

[51] Lunn DJ, Thomas A, Best N, Spiegelhalter D, BUGS: Bayesian Inference Using Gibbs Sampling. MRC Biostatistics Unit; 2023.

[52] Åsa M Johansson, Ueckert S, Plan EL, Hooker AC, Karlsson MO. Evaluation of bias, precision, robustness and runtime for estimation methods in NONMEM 7. Journal of Pharmacokinetics and Pharmacodynamics, 2014;41:223–238.

[53] Pradhan S, Song B, Lee J, Chae JW, Kim KI, Back HM, Han N, Kwon KI, Yun HY. Performance comparison of first-order conditional estimation with interaction and Bayesian estimation methods for estimating the population parameters and its distribution from data sets with a low number of subjects. BMC Medical Research Methodology, 2017;17.

[54] Plan EL, Maloney A, Mentré F, Karlsson MO, Bertrand J. Performance comparison of various maximum likelihood nonlinear mixed-effects estimation methods for dose-response models. AAPS Journal, 2012;14:420–432.

[55] Gibiansky L, Gibiansky E, Bauer R. Comparison of Nonmem 7.2 estimation methods and parallel processing efficiency on a target-mediated drug disposition model. Journal of Pharmacokinetics and Pharmacodynamics, 2012;39:17–35.

[56] Ette EI, Williams PJ. Population pharmacokinetics I: background, concepts, and models. The Annals of pharmacotherapy, 2004;38:1702–1706.

[57] Gabrielsson J, Weiner D. In: Reisfeld B, Mayeno AN, editors. Non-compartmental Analysis Totowa, NJ: Humana Press; 2012. p. 377–389.

[58] Proost JH. Combined proportional and additive residual error models in population pharmacokinetic modelling. European Journal of Pharmaceutical Sciences, 2017;109:S78–S82.

[59] Nguyen THT, Mouksassi MS, Holford N, Al-Huniti N, Freedman I, Hooker AC, John J, Karlsson MO, Mould DR, Ruixo JJP, Plan EL, Savic R, van Hasselt JGC, Weber B, Zhou C, Comets E, Mentré F. Model Evaluation of Continuous Data Pharmacometric Models: Metrics and Graphics. CPT: Pharmacometrics & systems pharmacology, 2017;6:87–109.

[60] Sanghavi K, Ribbing J, Rogers JA, Ahmed MA, Karlsson MO, Holford N, Chasseloup E, Ahamadi M, Kowalski KG, Cole S, Kerwash E, Wade JR, Liu C, Wang Y, Trame MN, Zhu H, Wilkins JJ. Covariate modeling in pharmacometrics: General points for consideration. CPT: Pharmacometrics & systems pharmacology, 2024;.

[61] Xu XS, Yuan M, Zhu H, Yang Y, Wang H, Zhou H, Xu J, Zhang L, Pinheiro J. Full covariate modelling approach in population pharmacokinetics: understanding the underlying hypothesis tests and implications of multiplicity. British journal of clinical pharmacology, 2018;84:1525–1534.

[62] Jonsson EN, Nyberg J. Using forest plots to interpret covariate effects in pharmacometric models. CPT: Pharmacometrics & systems pharmacology, 2024;.

[63] Wählby U, Jonsson EN, Karlsson MO. Comparison of Stepwise Covariate Model Building Strategies in Population Pharmacokinetic-Pharmacodynamic Analysis. AAPS PharmSci, 2002;4:27.

[64] Prague M, Lavielle M. SAMBA: A novel method for fast automatic model building in nonlinear mixed-effects models. CPT: Pharmacometrics and Systems Pharmacology, 2022;11:161–172.

[65] Ayral G, Abdallah JFS, Magnard C, Chauvin J. A novel method based on unbiased correlations tests for covariate selection in nonlinear mixed effects models: The COSSAC approach. CPT: Pharmacometrics and Systems Pharmacology, 2021;10:318–329.

[66] Dykstra K, Pugh R, Krause A. Visualization concepts to enhance quantitative decision making in drug development. Journal of clinical pharmacology, 2010;50:130S–139S.

[67] Sherwin CMT, Kiang TKL, Spigarelli MG, Ensom MHH. Fundamentals of Population Pharmacokinetic Modelling. Clinical Pharmacokinetics, 2012;51:573–590.

[68] Savic RM, Karlsson MO. Importance of shrinkage in empirical bayes estimates for diagnostics: problems and solutions. The AAPS journal, 2009;11:558–569.

[69] Zhang J, Yang Y, Ding J, Information criteria for model selection. John Wiley and Sons Inc; 2023.

[70] Karlsson MO, Savic RM. Diagnosing Model Diagnostics. Clinical Pharmacology & Therapeutics, 2007;82:17–20.

[71] Broeker A, Wicha SG. Assessing parameter uncertainty in small-n pharmacometric analyses: value of the log-likelihood profiling-based sampling importance resampling (LLP-SIR) technique. Journal of Pharmacokinetics and Pharmacodynamics, 2020;47:219–228.

[72] Fujita K, Okada K, Katahira K, The Fisher information matrix: A tutorial for calculation for decision making models. PsyArXiv; 2022.

[73] Jonsson EN, Nyberg J. A quantitative approach to the choice of number of samples for percentile estimation in bootstrap and visual predictive check analyses. CPT: Pharmacometrics & systems pharmacology, 2022;11:673–686.

[74] Hunt CA, Givens GH, Guzy S. Bootstrapping for Pharmacokinetic Models: Visualization of Predictive and Parameter Uncertainty. Pharmaceutical Research, 1998;15:690–697.

[75] Dosne AG, Bergstrand M, Karlsson MO. An automated sampling importance resampling procedure for estimating parameter uncertainty. Journal of Pharmacokinetics and Pharmacodynamics, 2017;44:509–520.

[76] Montgomery DC, Peck EA, Vining GG. Introduction to Linear Regression Analysis. Wiley Series in Probability and Statistics, 2012;.

[77] Hooker AC, Staatz CE, Karlsson MO. Conditional weighted residuals (CWRES): A model diagnostic for the FOCE method. Pharmaceutical Research, 2007;24:2187–2197.

[78] Oberg A, Davidian M. Estimating Data Transformations in Nonlinear Mixed Effects Models. Biometrics, 2004;56(1):65–72.

[79] Petersson KJF, Hanze E, Savic RM, Karlsson MO. Semiparametric distributions with estimated shape parameters. Pharmaceutical Research, 2009;26:2174–2185.

[80] Bergstrand M, Hooker AC, Wallin JE, Karlsson MO. Prediction-corrected visual predictive checks for diagnosing nonlinear mixed-effects models. AAPS Journal, 2011;13:143–151.

[81] Comets E, Brendel K, Mentré F. Computing normalised prediction distribution errors to evaluate nonlinear mixed-effect models: The npde add-on package for R. Computer Methods and Programs in Biomedicine, 2008;90:154–166.

[82] Nguyen THT, Comets E, Mentré F. Extension of NPDE for evaluation of nonlinear mixed effect models in presence of data below the quantification limit with applications to HIV dynamic model. Journal of Pharmacokinetics and Pharmacodynamics, 2012;39:499–518.

[83] Li X, Sale M, Nieforth K, Bigos KL, Craig J, Wang F, Feng K, Hu M, Bies R, Zhao L. pyDarwin: A Machine Learning Enhanced Automated Nonlinear Mixed-Effect Model Selection Toolbox. Clinical Pharmacology & Therapeutics, 2024;115(4):758–773.

[84] Chen X, Nordgren R, Belin S, Hamdan A, Wang S, Yang T, Huang Z, Carter SJ, Buatois S, Abrantes JA, Hooker AC, Karlsson MO. A fully automated tool for development of population pharmacokinetic models. CPT Pharmacometrics Systems Pharmacology, 2024;Published online ahead of print.

[85] Nordgren R, Ueckert S, Belin S, Yngman G, Carter S, Buatois S, Abrantes JA, Hooker AC, Karlsson MO, Pharmpy and assemblerr - Two novel tools to simplify the model building process in NONMEM. Annual Meeting of the Population Approach Group in Europe; 2021. www.page-meeting.org/?abstract=9656.

[86] Li X, Sale M, Nieforth K, Craig J, Wang F, Solit D, Feng K, Hu M, Bies R, Zhao L. pyDarwin machine learning algorithms application and comparison in nonlinear mixed-effect model selection and optimization. Journal of Pharmacokinetics and Pharmacodynamics, 2024;.

[87] Poweleit EA, Vinks AA, Mizuno T. Artificial Intelligence and Machine Learning Approaches to Facilitate Therapeutic Drug Management and Model-Informed Precision Dosing. Therapeutic Drug Monitoring, 2023;45:143–150.

[88] The European Medicines Agency. Guideline on reporting the results of population pharmacokinetic analyses Doc. Ref. CHMP/EWP/185990/06; 2007.

[89] Grasela TH, Dement CW, Kolterman OG, Fineman MS, Grasela DM, Honig P, Antal EJ, Bjornsson TD, Loh E. Pharmacometrics and the transition to model-based development. Clinical pharmacology and therapeutics, 2007;82:137–142.

[90] Frank T, Gastine S, Lindauer K, Speth H, Strougo A, Kovar A. LaTeX tutorial for the standardization and automation of population analysis reports. CPT: Pharmacometrics & systems pharmacology, 2021;10:1310–1322.

[91] Dykstra K, Mehrotra N, Tornøe CW, Kastrissios H, Patel B, Al-Huniti N, Jadhav P, Wang Y, Byon W. Reporting guidelines for population pharmacokinetic analyses. Journal of pharmacokinetics and pharmacodynamics, 2015;42:301–314.

[92] Okada A, Kariya M, Irie K, Okada Y, Hiramoto N, Hashimoto H, Kajioka R, Maruyama C, Kasai H, Hamori M, Nishimura A, Shibata N, Fukushima K, Sugioka N. Population pharmacokinetics of vancomycin in patients undergoing allogeneic hematopoietic stem-cell transplantation. Journal of Clinical Pharmacology, 2018;58:1140–1149.

[93] Shimamoto Y, Verstegen RHJ, Mizuno T, Schechter T, Allen U, Ito S. Population pharmacokinetics of vancomycin in paediatric patients with febrile neutropenia and augmented renal clearance: development of new dosing recommendations. Journal of Antimicrobial Chemotherapy, 2021;76:2932–2940.
