# Supplementary material for: Recommended approaches for integration of population pharmacokinetic modelling with precision dosing in clinical practice
Source: Br J Clin Pharmacol. 2024 Nov 21;91(4):1064–79. doi: 10.1111/bcp.16335 (PMC11992666; doi:10.1111/bcp.16335)
Supplement: Supplementary file 2 — DATA S2 Supporting Information. [file BCP-91-1064-s004.docx]

| **Summary** | | | | | | | | | |
| --- | --- | --- | --- | --- | --- | --- | --- | --- | --- |
| Model name: |  | | | Population type*: | | |  | | |
| Drug*: |  | | | Model type: | | |  | | |
| Administration route*: |  | | | No. compartments*: | | |  | | |
| **Publication** | | | | | | | | | |
| Title: |  | | | | | | | | |
| Author(s): |  | | | | | | | | |
| Other info: |  | | | | | | | | |
| Publication URL*: |  | | | | | | | | |
| **Source Study & Dataset** | | | | | | | | | |
| Source study: |  | | | | | | | | |
| Inclusion criteria: |  | | | | | | | | |
| Patient disease(s): |  | | | | | | | | |
| Drug dosing units*: |  | | | Number of patients: | | |  | | |
| Plasma conc. units*: |  | | | No. samples: | | |  | | |
| Notes/further info: |  | | | | | | | | |
| **Patient Characteristics** (adjust as appropriate) | | | | | | | | | |
| **Continuous covariate** | | **Units** | **Median** | | **Mean** | **SD** | | **Mode** | **Range** |
| Age | |  |  | |  |  | |  |  |
| Weight | |  |  | |  |  | |  |  |
| Body surface area | |  |  | |  |  | |  |  |
| … | |  |  | |  |  | |  |  |
| **Categorical covariate** | | **Feature** | | | | **Count (%)** | | | |
| Sex | |  | | | |  | | | |
| Sex | |  | | | |  | | | |
| … | |  | | | |  | | | |
| Notes/further info: | |  | | | | | | | |

| **Final Model Structure** | | | |  |
| --- | --- | --- | --- | --- |
| Compartments: |  | | |  |
| Elimination (e.g. 1^st^ order): |  | | |  |
| Estimation algorithm: |  | Log-transformed? |  |  |
| BLLOQ handling method: |  | Dose compartment: |  |  |
| All covariates tested: |  | | |  |
| Covariate inclusion method: |  | | |  |
| Covariates included*: |  | | |  |
| 'Typical' patient for scaling: |  | | |  |
| Graphical representation / schematic |  | | |  |
| Equations |  | | |  |
|  |  |  |  |  |
|  |  |  |  |  |
|  |  |  |  |  |
|  |  |  |  |  |
|  |  |  |  |  |
| Notes/further info: |  | | |  |

| **Parameter Estimates*** (adjust as appropriate) | | | | | |
| --- | --- | --- | --- | --- | --- |
| PK parameter (units) | Value | 95% CI | CV% | RSE | Shrinkage |
| **Fixed effects** | | | | | |
| CL (L/h) |  |  |  |  |  |
| V1 (L) |  |  |  |  |  |
| Q (L/h) |  |  |  |  |  |
| V2 (L) |  |  |  |  |  |
| … |  |  |  |  |  |
| **Between-subject variability (inter-individual variability)** | | | | | |
| BSV on CL |  |  |  |  |  |
| BSV on V1 |  |  |  |  |  |
| … |  |  |  |  |  |
| **Between-occasion variability (inter-occasion variability)** | | | | | |
| BOV on CL |  |  |  |  |  |
| … |  |  |  |  |  |
| **Residual error** | | | | | |
| Proportional error |  |  |  |  |  |
| Additive error |  |  |  |  |  |
| Notes/further info: |  | | | | |

** required as a minimum for model replication*

| **Model Evaluation Metrics** | | | | |
| --- | --- | --- | --- | --- |
| Does the model publication provide the following? | | | | |
| Visual predictive check (VPC) plot(s): | |  | Example plasma conc. profiles: |  |
| Other goodness-of-fit plots: | |  | Simulated plasma conc. profiles: |  |
| Notes/further info: |  | | | |

**Figures and diagnostic plots**

Paste diagnostic plots etc from original publication here and compare to those generated in validation.
