# Supplementary material for: Recommended approaches for integration of population pharmacokinetic modelling with precision dosing in clinical practice
Source: Br J Clin Pharmacol. 2024 Nov 21;91(4):1064–79. doi: 10.1111/bcp.16335 (PMC11992666; doi:10.1111/bcp.16335)
Supplement: Supplementary file 3 — DATA S3 Supporting Information. [file BCP-91-1064-s003.docx]

| **Summary** | | | | | | | | | | | |
| --- | --- | --- | --- | --- | --- | --- | --- | --- | --- | --- | --- |
| Model name: | Vancomycin_example1 | | | | Population type*: | | | | Adults | | |
| Drug*: | Vancomycin | | | | Model type: | | | | PopPK | | |
| Administration route*: | Intravenous | | | | No. compartments*: | | | | 2 | | |
| **Publication** | | | | | | | | | | | |
| Title: | Population pharmacokinetics of vancomycin in patients undergoing allogeneic hematopoietic stem-cell transplantation | | | | | | | | | | |
| Author(s): | Akira Okada, Misato Kariya, Kei Irie, Yutaka Okada, Nobuhiro Hiramoto, Hisako Hashimoto, Ryosuke Kajioka, Chika Maruyama, Hidefumi Kasai, Mami Hamori, Asako Nishimura, Nobuhito Shibata, Keizo Fukushima, and Nobuyuki Sugioka | | | | | | | | | | |
| Other info: | Journal of Clinical Pharmacology, 2018, 58(9), 1140-1149 | | | | | | | | | | |
| Publication URL*: | https://accp1.onlinelibrary.wiley.com/doi/10.1002/jcph.1106 | | | | | | | | | | |
| **Source Study & Dataset** | | | | | | | | | | | |
| Source study: | Consenting patients at Institute of Biomedical Research and Innovation Hospital, Osaka, Japan | | | | | | | | | | |
| Inclusion criteria: | Patients undergoing allo-HSCT receiving preventative treatment with vancomycin. Excluded acute kidney injury in last 7 days (see paper) | | | | | | | | | | |
| Patient disease(s): | Allo-HSCT, receiving treatment to prevent bacterial infection | | | | | | | | | | |
| Drug dosing units*: | g | | | | Number of patients: | | | | 95 (75 for model development, 20 for external validation) | | |
| Plasma conc. units*: | µg/mL (mg/L) | | | | No. samples: | | | | 285 (227 for model development, 58 for validation) | | |
| Notes/further info: | Study conducted in Japan | | | | | | | | | | |
| **Patient Characteristics** (adjust as appropriate) | | | | | | | | | | | |
| **Continuous covariate** | | **Units** | | **Median** | | **Mean** | **SD** | | | **Mode** | **Range** |
| Vancomycin daily dose | | g | | 2.4 | | - | - | | | - | 1.0-4.5 |
| Age | | years | | 49 | | - | - | | | - | 17-69 |
| Body weight | | kg | | 59.4 | | - | - | | | - | 39.4-104.5 |
| Body surface area | | m^2^ | | 1.66 | | - | - | | | - | 1.34-2.29 |
| Hematocrit | | % | | 24.2 | | - | - | | | - | 17.9-35.5 |
| Albumin | | g/dL | | 3.0 | | - | - | | | - | 1.8-3.9 |
| C-reactive protein | | mg/dL | | 4.6 | | - | - | | | - | 0.2-25.7 |
| Total bilirubin | | mg/dL | | 0.5 | | - | - | | | - | 0.1-3.7 |
| Blood urea nitrogen | | mg/dL | | 15 | | - | - | | | - | 5-49 |
| Serum creatinine | | mg/dL | | 0.62 | | - | - | | | - | 0.29-1.63 |
| Creatinine clearance | | mL/min/1.73 m^2^ | | 113 | | - | - | | | - | 47-253 |
| **Categorical covariate** | | | **Feature** | | | | | **Count (%)** | | | |
| Sex | | | Male | | | | | 49 (65.3%) | | | |
| Sex | | | Female | | | | | 26 (44.7%) | | | |
| Conditioning of transplantation | | | Myeloablative conditioning | | | | | 60 (80.0%) | | | |
| Conditioning of transplantation | | | Reduced-intensity conditioning | | | | | 15 (20.0%) | | | |
| Hematologic disease | | | Acute myelocytic leukemia | | | | | 29 (38.7%) | | | |
| Hematologic disease | | | Acute lymphocytic leukemia | | | | | 13 (17.3%) | | | |
| Hematologic disease | | | Myelodysplastic syndrome | | | | | 6 (8.0%) | | | |
| Hematologic disease | | | Adult T-cell leukemia | | | | | 5 (6.7%) | | | |
| Hematologic disease | | | Diffuse large B-cell lymphoma | | | | | 4 (5.3%) | | | |
| Hematologic disease | | | Chronic myelocytic leukemia | | | | | 4 (5.3%) | | | |
| Hematologic disease | | | Other | | | | | 14 (18.7%) | | | |
| Notes/further info: | |  | | | | | | | | | |

| **Final Model Structure** | | | |  |
| --- | --- | --- | --- | --- |
| Compartments: | Central, peripheral | | |  |
| Elimination (e.g. 1^st^ order): | First-order | | |  |
| Estimation algorithm: | FOCE | Log-transformed? | No |  |
| BLLOQ handling method: | Not specified | Dose compartment: | Central |  |
| All covariates tested: | Sex, age, BW, BSA, albumin, Hct, RBC, WBC, T-BIL, CRP, BUN, SCr, and CLCr, underlying hematologic disease including acute myelocytic leukemia, acute lymphocytic leukemia, adult T-cell leukemia, myelodysplastic syndrome, and diffuse large B-cell lymphoma; concomitant drugs including cyclosporine, tacrolimus, and antimicrobial agents; and allo-HSCT conditioning (myeloablative or reduced-intensity conditioning) | | |  |
| Covariate inclusion method: | Stepwise forward/back | | |  |
| Covariates included*: | Body weight on V1, creatinine clearance on CL | | |  |
| 'Typical' patient for scaling: | Body weight 59.4 kg; creatinine clearance 113 (mL/min/1.73m^2^) | | |  |
| Graphical representation / schematic | Not provided | | |  |
| Equations | 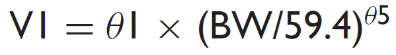  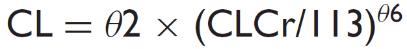  Proportional error  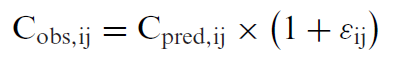 | | |  |
|  |  |  |  |  |
|  |  |  |  |  |
|  |  |  |  |  |
|  |  |  |  |  |
|  |  |  |  |  |
| Notes/further info: |  | | |  |

| **Parameter Estimates*** (adjust as appropriate) | | | | | |
| --- | --- | --- | --- | --- | --- |
| PK parameter (units) | Value | 95% CI | CV% | RSE% | Shrinkage |
| **Fixed effects** | | | | | |
| CL (L/h) | 4.25 | - | - | 2.7 | - |
| V1 (L) | 39.2 | - | - | 5.01 | - |
| Q (L/h) | 1.95 | - | - | 8.26 | - |
| V2 (L) | 56.1 | - | - | 24.5 | - |
| CLCr on CL | 0.70 | - | - | 10.5 | - |
| BW on V1 | 0.78 | - | - | 9.79 | - |
| **Between-subject variability (inter-individual variability)** | | | | | |
| IIV on CL | 25.2 | - | - | 15.9 | - |
| IIV on V1 | 14.2 | - | - | 25.2 | - |
| IIV on V2 | 66.9 | - | - | 23.1 | - |
| **Residual error** | | | | | |
| Proportional error | 17.2 | - | - | 9.14 | - |
| Notes/further info: |  | | | | |

** required as a minimum for model replication*

| **Model Evaluation Metrics** | | | | |
| --- | --- | --- | --- | --- |
| Does the model publication provide the following? | | | | |
| Visual predictive check (VPC) plot(s): | | Yes (supplementary S1) | Example plasma conc. profiles: | Yes (fig1) |
| Other goodness-of-fit plots: | | Yes (fig2) | Simulated plasma conc. profiles: | Yes (fig3) |
| Notes/further info: |  | | | |
