# Supplementary material for: Recommended approaches for integration of population pharmacokinetic modelling with precision dosing in clinical practice
Source: Br J Clin Pharmacol. 2024 Nov 21;91(4):1064–79. doi: 10.1111/bcp.16335 (PMC11992666; doi:10.1111/bcp.16335)
Supplement: Supplementary file 4 — DATA S4 Supporting Information. [file BCP-91-1064-s002.docx]

| **Summary** | | | | | | | | | |
| --- | --- | --- | --- | --- | --- | --- | --- | --- | --- |
| Model name: | Vancomycin_example2 | | | Population type*: | | | Paediatric | | |
| Drug*: | Vancomycin | | | Model type: | | | PopPK | | |
| Administration route*: | Intravenous | | | No. compartments*: | | | 2 | | |
| **Publication** | | | | | | | | | |
| Title: | Population pharmacokinetics of vancomycin in paediatric patients with febrile neutropenia and augmented renal clearance: development of new dosing recommendations | | | | | | | | |
| Author(s): | Yuko Shimamoto, Ruud H. J. Verstegen, Tomoyuki Mizuno, Tal Schechter, Upton Allen and Shinya Ito | | | | | | | | |
| Other info: | Journal of Antimicrobial Chemotherapy, 2021, 76, 2932-2940 | | | | | | | | |
| Publication URL*: | https://academic.oup.com/jac/article/76/11/2932/6364109 | | | | | | | | |
| **Source Study & Dataset** | | | | | | | | | |
| Source study: | Patients at Toronto Hospital for Sick Children, Canada. See paper | | | | | | | | |
| Inclusion criteria: | Prior vancomycin treatment for febrile neutropenia received within 2 months of hematopoietic stem cell transplantation (HSCT). Under 18 years old. Patients on renal replacement therapy at time of treatment were excluded. | | | | | | | | |
| Patient disease(s): | Various, with febrile neutropenia following HSCT | | | | | | | | |
| Drug dosing units*: | mg/kg/day | | | Number of patients: | | | 165 | | |
| Plasma conc. units*: | µg/mL (mg/L) | | | No. samples: | | | 276 | | |
| Notes/further info: | Study conducted in Canada. Many samples obtained at trough concentrations | | | | | | | | |
| **Patient Characteristics** (adjust as appropriate) | | | | | | | | | |
| **Continuous covariate** | | **Units** | **Median** | | **Mean** | **SD** | | **Mode** | **Inter-quartile range** |
| Weight | | kg | 20.7 | | - | - | | - | 13.5-46.5 |
| Body temperature | | °C | 38.6 | | - | - | | - | 37.5-39.4 |
| Heart rate | | bpm | 136 | | - | - | | - | 116-148 |
| Respiratory rate | | bpm | 28 | | - | - | | - | 24-36 |
| Neutrophil count | | /mm^3^ | 10 | | - | - | | - | 0-30 |
| Serum creatinine | | mg/dL | 0.34 | | - | - | | - | 0.25-0.45 |
| eGFR | | mL/min/1.73m^2^ | 143 | | - | - | | - | 117-168 |
| Serum total bilirubin | | µmol/L | 6 | | - | - | | - | 3-10 |
| Serum albumin | | g/L | 31 | | - | - | | - | 28-35 |
| **Categorical covariate** | | **Feature** | | | | **Count (%)** | | | |
| Sex | | Male | | | | 93 (56.4%) | | | |
| Sex | | Female | | | | 72 (43.6%) | | | |
| Age distribution* | | 3 months to <6 months | | | | 3 (1.8%) | | | |
| Age distribution* | | 6 months to <1 year | | | | 11 (6.7%) | | | |
| Age distribution* | | 1 year to <2 years | | | | 14 (8.5%) | | | |
| Age distribution* | | 2 years to <6 years | | | | 50 (30.3%) | | | |
| Age distribution* | | 6 years to <12 years | | | | 36 (21.8%) | | | |
| Age distribution* | | 12 years to 18 years | | | | 51 (30.9%) | | | |
| Diagnosis | | Leukaemia and lymphoma | | | | 75 (45.5%) | | | |
| Diagnosis | | Solid tumour | | | | 29 (17.6%) | | | |
| Diagnosis | | Haematological disorder | | | | 22 (13.3%) | | | |
| Diagnosis | | Brain tumour | | | | 17 (10.3%) | | | |
| Diagnosis | | Immunodeficiency | | | | 14 (8.5%) | | | |
| Diagnosis | | Metabolic disease | | | | 8 (4.8%) | | | |
| Transplant source | | Bone marrow | | | | 82 (49.7%) | | | |
| Transplant source | | Peripheral blood stem cell | | | | 36 (21.8%) | | | |
| Transplant source | | Cord blood stem cell | | | | 47 (28.5%) | | | |
| Distribution of eGFR | | ≤59 mL/min/1.73m^2^ | | | | 1 (0.6%) | | | |
| Distribution of eGFR | | 60-79 mL/min/1.73m^2^ | | | | 8 (4.8%) | | | |
| Distribution of eGFR | | 80-99 mL/min/1.73m^2^ | | | | 14 (8.5%) | | | |
| Distribution of eGFR | | 100-119 mL/min/1.73m^2^ | | | | 26 (15.8%) | | | |
| Distribution of eGFR | | 120-139 mL/min/1.73m^2^ | | | | 27 (16.4%) | | | |
| Distribution of eGFR | | 140-159 mL/min/1.73m^2^ | | | | 33 (20.0%) | | | |
| Distribution of eGFR | | 160-179 mL/min/1.73m^2^ | | | | 28 (17.0%) | | | |
| Distribution of eGFR | | 180-199 mL/min/1.73m^2^ | | | | 9 (5.5%) | | | |
| Distribution of eGFR | | ≥200 mL/min/1.73m^2^ | | | | 19 (11.5%) | | | |
| Notes/further info: | | * continuous, but displayed as categorical | | | | | | | |

| **Final Model Structure** | | | |  |
| --- | --- | --- | --- | --- |
| Compartments: | Central, peripheral | | |  |
| Elimination (e.g. 1^st^ order): | Not stated | | |  |
| Estimation algorithm: | Not stated | Log-transformed? | No |  |
| BLLOQ handling method: | Not stated | Dose compartment: | Central |  |
| All covariates tested: | Body weight (WT), age (maturation function), serum creatinine, eGFR, systemic inflammatory response syndrome (body temperature, heart rate, respiratory rate), primary diagnosis, transplant type | | |  |
| Covariate inclusion method: | Not stated | | |  |
| Covariates included*: | WT allometric scaling on all; post-menstrual age sigmoidal Emax/Hill eqn on CL; eGFR on CL; Fever status on CL | | |  |
| 'Typical' patient for scaling: | WT 70kg; eGFR 120 mL/min/1.73m^2^ | | |  |
| Graphical representation / schematic |  | | |  |
| Equations | 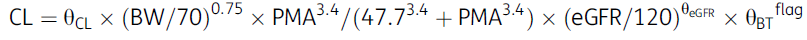 | | |  |
|  |  |  |  |  |
|  |  |  |  |  |
|  |  |  |  |  |
|  |  |  |  |  |
|  |  |  |  |  |
| Notes/further info: | V1, V2 and Q fixed to previously reported values from large-scale study. See paper.  If BT ≥38C then BT on CL effect is included | | |  |

| **Parameter Estimates*** (adjust as appropriate) | | | | | |
| --- | --- | --- | --- | --- | --- |
| PK parameter (units) | Value | 95% CI | CV% | RSE% | Shrinkage |
| **Fixed effects** | | | | | |
| CL (L/h) | 5.94 | - | - | 3.0 | - |
| V1 (L/70kg) | 39.9 | - | - | - | - |
| Q (L/h) | 3.85 | - | - | - | - |
| V2 (L/70kg) | 37.8 | - | - | - | - |
| WT on CL Q | 0.75 | - | - | - | - |
| WT on V | 1.0 | - | - | - | - |
| TM_50_ | 47.7 | - | - | - | - |
| Hill | 3.4 | - | - | - | - |
| GFR on CL | 0.626 | - | - | 11 | - |
| Body temperature on CL | 1.12 | - | - | 4 | - |
| **Between-subject variability (inter-individual variability)** | | | | | |
| IIV on CL | 0.052 | - | - | 15 | - |
| **Residual error** | | | | | |
| Proportional error | 0.0855 | - | - | 18 | - |
| Notes/further info: | TM_50_ = maturation half-time | | | | |

** required as a minimum for model replication*

| **Model Evaluation Metrics** | | | | |
| --- | --- | --- | --- | --- |
| Does the model publication provide the following? | | | | |
| Visual predictive check (VPC) plot(s): | | Yes (fig2) | Example plasma conc. profiles: | No |
| Other goodness-of-fit plots: | | Yes (fig1) | Simulated plasma conc. profiles: | Yes (in VPC) |
| Notes/further info: |  | | | |
